# An Overview of the Crosstalk Dysregulation Between miRNAs and Signaling Pathways in Cancer: A Recent Perspective on Regulatory Networks

**DOI:** 10.3390/ijms272110046

**Published:** 2026-11-09

**Authors:** Abdulaziz A. Aloliqi, Abdullah M. Alnuqaydan, Amjad Ali Khan

**Affiliations:** Department of Basic Health Sciences, College of Applied Medical Sciences, Qassim University, Buraydah 51452, Saudi Arabia; aaalieky@qu.edu.sa (A.A.A.); ami.alnuqaydan@qu.edu.sa (A.M.A.)

**Keywords:** miRNA, signaling pathways, crosstalk, cancer, oncomiRs, regulatory network, clinical trials

## Abstract

The discovery of microRNAs (miRNAs) has revolutionized the concept of posttranscriptional gene regulation. miRNAs play a significant role in regulating key signaling pathways for the maintenance of cellular homeostasis under physiological conditions. However, the cancerous state of the cell leads to the dysregulation of miRNA expression and its proper function due to genetic alterations, epigenetic remodeling, unusual transcriptional control, and defects in its biogenesis. A proper crosstalk between the intracellular signaling pathways and the miRNAs is significantly disrupted in cancer cells, leading to rewiring of oncogenic networks. In this review, an updated mechanism of miRNA biosynthesis and its dysregulation at each regulatory layer is elaborated. The functional consequences of defective miRNA biogenesis, including loss of tumor-suppressive miRNAs, amplification of oncogenic signaling, and acquisition of therapy resistance, are discussed. The functional consequences of the downregulated tumor-suppressive miRNA network and the upregulation of oncogenic signaling in core signaling pathways such as PI3K/AKT/mTOR, MAPK/RAS/ERK, Wnt/β-catenin, TGF-β/SMAD, JAK/STAT3, and Notch signaling are discussed. Moreover, the outcome of miRNA-signaling pathways, dysregulated crosstalk during epithelial–mesenchymal transition, metastasis, maintenance of cancer stem cells, and chemoresistance is highlighted. Furthermore, current challenges in targeting tumor-suppressive miRNAs and oncomiRs in cancer cells are highlighted. Besides this, the pioneering role of multiomics for the investigation of miRNA-regulated signaling is described. Collectively, this review features the understanding of miRNA-signaling crosstalk and its translational potential in precision oncology.

## 1. Introduction

MicroRNAs (miRNAs) are a highly conserved class of endogenous, short noncoding RNAs typically ~20–25 nucleotides in length that function as key regulators of gene expression at the posttranscriptional level [1,2]. miRNAs regulate normal homeostasis, including cell proliferation, differentiation, apoptosis, and stress response [3]. They exert their regulatory activity by base pairing with complementary sequences in the 3′ untranslated regions (3′UTRs) of target messenger RNAs (mRNAs), leading either to mRNA degradation or inhibition of translation via the RNA-induced silencing complex (RISC) machinery [4]. This canonical mode of action enables miRNAs to control the expression of a large portion of the human genome and fine-tune diverse biological processes [5].

Signaling pathways play a significant role in maintaining the normal physiology of a cell and miRNAs help to regulate crucial nodes within complex signaling networks. However, in pathological states including cancer, the expression profile of different miRNAs is severely altered [6]. Different types of genetic and epigenetic alterations involve defective miRNA biogenesis and dysregulation of transcription factors (TFs) [7]. These abnormal cellular changes can shift the balance of miRNA-mediated regulation such that key signaling pathways become persistently activated or inappropriately suppressed, contributing to the hallmarks of cancer including sustained proliferative signaling, evasion of apoptosis, enhanced migration and invasion, metastatic dissemination, and therapy resistance [8,9].

Dysregulated miRNAs in cancer may act as oncogenic miRNAs (oncomiRs) by repressing tumor-suppressor targets or tumor-suppressor miRNAs by targeting oncogenic components; either direction of change can disrupt normal signaling pathway dynamics [10]. These deviations alter the crosstalk between miRNAs and signaling networks by rewiring feedback and feed-forward loops, creating resilient signaling states that promote malignant transformation [6,11].

The integration of miRNAs into canonical signaling pathways, such as PI3K (Phosphoinositide 3-Kinase)/AKT (Protein Kinase B)/mTOR (mechanistic Target of Rapamycin), Wnt (Wingless and Integration 1)/β-catenin, MAPK (Mitogen-Activated Protein Kinase)/ERK (Extracellular Signal-Regulated Kinase), TGF-β (Transforming Growth Factor-β), and Notch signaling, underscores their ability to coordinate multipathway responses and sculpt the cancer signaling landscape [12,13]. Dysregulation of these miRNA-signaling networks not only fuels oncogenesis but also confers phenotypic plasticity that underlies metastasis and resistance to conventional therapies [11,14].

Consequently, a systems-based framework that treats miRNAs and signaling pathways as integrated regulatory networks is necessary for comprehensively understanding cancer biology [15]. Such an approach may also widen the therapeutic opportunities, including the modulation of miRNAs located at critical network nodes to restore abnormal signaling patterns and the development of strategies that interrupt disease-promoting feedback circuits [16,17].

## 2. miRNA Biogenesis and Functional Regulation in Cancer

The biogenesis of miRNAs is tightly controlled under normal physiology, but this multistep pathway is often disrupted in cancer [15]. Tumor-suppressive miRNAs and oncomiRs influence miRNA biogenesis by regulating key processing enzymes, thereby altering the maturation of other miRNAs. Understanding this process and its dysregulation is crucial because alterations at any stage of biogenesis can profoundly affect miRNA abundance, target regulation, and downstream signaling networks that drive tumorigenesis and progression [17].

### 2.1. Overview of miRNA Biogenesis

The canonical miRNA biogenesis pathway is a complex, well-coordinated, and multistep process starting with the transcription of the miRNA gene, mostly by RNA polymerase II [4]. miRNA biogenesis involves multiple processes including primary miRNA (pri-miRNA) transcript formation, processing of pri-miRNA into precursor miRNA (pre-miRNA) and its export from the nucleus to the cytoplasm, and processing of pre-miRNA into mature miRNA. Each step is regulated by different factors, and any disruption of these regulatory factors significantly changes miRNA abundance, target specificity, and regulatory potential, promoting cancer initiation, progression, and therapeutic resistance [18].

**Figure 1:**
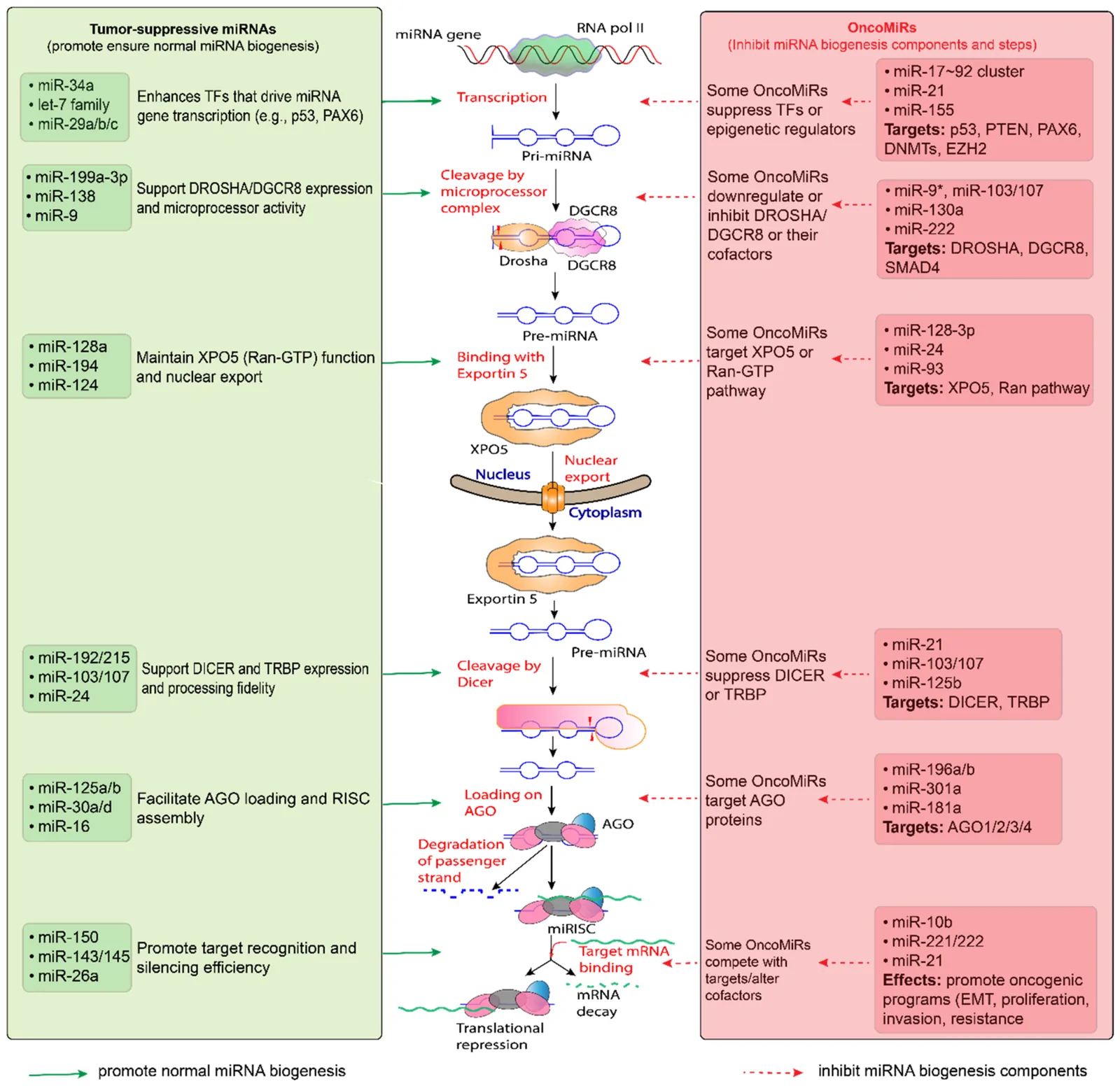
Canonical miRNA biogenesis pathway. Tumor-suppressive miRNAs help in normal pathway execution, while oncomiRs dysregulate this pathway at different steps. Abbreviations: AGO, Argonaute; DICER, Dicer endoribonuclease; DNMTs, DNA Methyltransferases; DROSHA/DGCR8, Drosha (Drosha ribonuclease III)/DGCR8 (DiGeorge Syndrome Critical Region 8); EMT, Epithelial–Mesenchymal Transition; EZH2, Enhancer of Zeste Homolog 2; PAX6, Paired Box 6; PTEN, Phosphatase and Tensin Homolog; RISC, RNA-Induced Silencing Complex; SMAD4, SMAD Family Member 4; TFs, Transcription Factors; TRBP, TAR RNA-Binding Protein; XPO5, Exportin 5.

The pri-miRNA is a hairpin-shaped noncoding RNA typically formed as ~1000 nucleotides long, 5′-capped, and 3′-polyadenylated transcripts and its expression is regulated by chromatin organization and epigenetic mechanisms [19] (Figure 1). Besides this, some oncogenic and tumor-suppressive TFs such as tumor protein 53 (p53), myelocytomatosis oncogene (MYC), and nuclear factor kappa-light-chain-enhancer of activated B cells (NF-κB) directly regulate some miRNAs [20]. The processing of pri-miRNA into its precursor form (pre-miRNA), which is hairpin-shaped and typically ~70–80 nucleotides long, is performed by the microprocessor complex, consisting of RNase III enzyme Drosha Ribonuclease III (DROSHA) and DiGeorge Syndrome Critical Region 8 (DGCR8). It has been reported that miR-122–pri-miR-21 interaction is particularly interesting because it regulates the DROSHA processing step without directly targeting the DROSHA gene. In contrast, miR-103/107, miR-221/222, and miR-29a act by targeting DICER1 mRNA. These represent two distinct mechanisms of regulating miRNA biogenesis [21].

This stage constitutes a key regulatory checkpoint, because its efficiency and selectivity are influenced by supplementary RNA-binding proteins and signaling-dependent cofactors [18]. Proteins such as p68/p72 helicases, Small Mothers Against Decapentaplegic homologs (SMADs), and heterogeneous nuclear ribonucleoproteins dynamically modulate pri-miRNA processing in response to the activation of pathways such as MAPK and TGF-β signaling [4]. Consequently, changes in the abundance, subcellular localization, or structural integrity of microprocessor components can selectively alter miRNA production in a context-specific manner [4].

Further processing of the pre-miRNA takes place in the cytoplasm following its transport through the nuclear membrane by exportin-5 (XPO5)-Ran GTP complex [18]. Following cytoplasmic import, pre-miRNA is processed by DICER, an RNase III enzyme, to form a mature ~20–22-nucleotide miRNA/miRNA duplex. Any dysregulation of DICER affects the pre-miRNA processing, leading to the formation of distinct miRNA isoforms (isomirs), usually seen in cancer cells [4,22]. It has been demonstrated that miR-103/107 targets DICER, reducing the expression of metastasis-suppressing miRNAs, including members of the miR-200 family, and facilitating metastatic progression [23].

Out of this miRNA/miRNA duplex, one strand acts as the passenger strand, while the other complementary strand acts as the mature or guide strand [24]. The duplex is further unwound, and the mature strand is incorporated into the argonaute (AGO)-containing RNA-induced silencing complex (RISC) and helps it to target the mRNA (Figure 1).

The miRNA-target mRNA interaction is mediated by a seed region (~6 to 8 nucleotides), located at the 5′-end of the miRNA through Watson–Crick base pairing. This interaction leads to the translational repression of mRNA, if the complementarity is imperfect, or can result in mRNA degradation if perfect complementarity occurs [25].

### 2.2. miRNA Biogenesis Dysregulation in Cancer

It is now well established from different studies that there is a strong link between dysregulated miRNA biogenesis and cancer [15,26]. As miRNAs are the key integrators of posttranscriptional control, these molecules significantly contribute to the initiation, progression, and resistance of cancer and to its therapeutic response [15]. miRNAs behave either as tumor-suppressive entities or as oncogenic factors (oncomiRs). The chromatin organization and aberrant expression of TFs also dysregulate the expression of miRNAs. The oncogenic miRNAs are significantly expressed by oncogenic drivers such as MYC, RAS, and NF-κB signaling, and this also leads to downregulation of tumor-suppressive miRNA expression [27].

Different miRNAs play a significant role in miRNA biogenesis and any deviated expression of DROSHA, DGCR8, XPO5, and DICER leads to diverse tumor formation [16,17]. Under normal conditions in a cell, some tumor-suppressive miRNAs (miR-34a, let-7 family, and miR-29) enhance the expression of TFs that drive normal miRNA transcription. However, some well-known oncomiRs (miR-17-92, miR-21, miR-155) target p53 and PTEN and suppress some TFs at the transcriptional level (Figure 1).

OncomiRs can also target, downregulate, or inhibit the DROSHA/DGCR8 complex and their cofactors. In addition, oncomiRs may also target or reduce the expression of XPO5-RanGTP, DICER, and AGO proteins and can compete with target mRNA binding [4] (Figure 1). All these adaptive changes promote tumorigenesis by enhancing the phenotypic plasticity, epithelial–mesenchymal transition (EMT), and therapeutic resistance [28]. The canonical pathway of miRNA biogenesis, its normal execution with the help of tumor-suppressive miRNAs, and its dysregulation by different oncomiRs at different stages are briefly illustrated in Figure 1.

### 2.3. miRNA Biogenesis Dysregulation and Its Functional Consequences

Although miRNA biogenesis is a tightly regulated process, its defective biogenesis affects almost the entire homeostasis, reshaping the whole cellular architecture [15]. Many elements of the PI3K/AKT, MAPK, Wnt/β-catenin, and Janus Kinase (JAK)/Signal Transducer and Activator of Transcription (STAT) pathways are derepressed when tumor-suppressive miRNA networks are attenuated [29]. Phenotypic heterogeneity, pathway cross-activation, and signaling noise are all increased by this loss of coordinated repression [30]. The cellular growth and survival cues are further reinforced by the selective preservation or augmentation of oncogenic miRNAs.

Every cellular process is regulated by miRNAs, including transcription of cellular junction proteins, EMT, cancer cell migration and invasion, stemness, angiogenesis, and tumor microenvironment (TME) [31]. The transcription of cellular junction proteins is also regulated by miRNAs, and these proteins are important for signaling cascades and growth [32]. The disruption of cellular junctions leads to metastasis; e.g., zonula occludens-1 (ZO-1), a significant component of the tight junction, is regulated by several miRNAs. As reported, ZO-1 is inhibited by miR-105, as this miRNA regulates the metastasis of breast cancer [33]. In addition, metastasis of cancer cells is induced by overexpression of miR-105 in other organs like the liver [33].

Zinc finger E-box-binding protein TFs such as ZEB1 and ZEB2 mostly control EMT and promote upregulation of vimentin and N-cadherin [34]. The miR-200 family, miR-124, and miR-429 regulate ZEB1 and ZEB2 and metastasis in liver, pancreatic [35], colorectal [34], breast [36], lung [37], and gastric cancers [38]. Furthermore, significant downregulation of miR-132-mediated ZEB2 has been reported in colorectal cancer (CRC) [39]. In CRC, the expression of miR-186-5p is downregulated and its overexpression suppresses EMT by directly targeting ZEB1; similarly, miR-215 directly targets ZEB2 [40,41].

The first miRNA linked to metastases in patients with advanced breast cancer was miR-10b [42]. By controlling homeobox D10 (HOXD10), miR-10b encourages migration and invasion in a variety of cancer types, including gastric, breast, and colorectal malignancies. In breast cancer, downregulation of miR-150 and overexpression of miR-96, miR-135a, and miR-21 promote migration and invasion. Similarly, oncogenic miR-96 targets miR-135a and protein tyrosine phosphatase, nonreceptor 9 (PTPN9) in this type of cancer [43]. In breast cancer, miR-135a promotes cell migration and invasion by directly targeting and suppressing HOXA10 expression. Similarly, the oncogenic miR-21-5p promotes breast cancer progression and metastasis by suppressing the expression of programmed cell death protein 4 (PDCD4), a tumor suppressor [44,45]. Breast cancer has abnormal expression of the tumor suppressor miR-150, which targets high mobility group AT-hook 2 (HMGA2) and encourages migration and invasion [46].

miRNAs control important aspects of tumor-mediated angiogenesis and metastatic growth. In breast cancer, stemness-related genes are targeted by downregulation of miR-200c, and overexpression of miR-30c influences the formation of secondary tumors [47]. The overexpression of vascular endothelial growth factor (VEGF) caused by oncogene miR-9-mediated suppression of E-cadherin increases angiogenesis, which is necessary for the development of secondary tumors [48].

miRNAs play a significant role in the development of metastases by mediating the interaction between the TME and tumor cells. Key elements of the TME are tumor-associated macrophages (TAMs), which are controlled by miRNAs to display protumor activities. For instance, highly amplified miR-301a in pancreatic cancer cells promotes the metastasis of pancreatic cancer cells by inducing M2 macrophage polarization through the PTEN/PI3K signaling pathway [49]. Furthermore, miR-130b is frequently upregulated in gastric cancer and has been implicated in tumor progression by promoting cancer cell proliferation and inhibiting apoptosis [50].

## 3. Principles of miRNA-Signaling Pathway Crosstalk

Given the abundance and diversity of miRNAs, they can target TFs, enzymes, and signaling proteins. miRNAs and their targets create a complex regulatory network intertwined with other cellular networks including signal-transduction networks, as evidenced by the diversity and number of miRNA targets, which offer an incredible level of combinatorial possibilities [51]. It is unknown, nevertheless, if and how miRNAs might coordinate their control of cellular signaling networks, as well as how control of these networks might support miRNAs’ biological roles. miRNA-signaling interactions are intrinsically multidirectional and network-based, involving reciprocal regulation, feedback and feed-forward loops, and hierarchical regulatory mechanisms, in contrast to linear signaling cascades [52]. This architecture allows cells to adjust signal amplitude, length, and integration in response to internal and external signals, but any disruption of it leads to malignant network rewiring.

### 3.1. Bidirectional Regulation Between miRNA and Signaling Pathways

By targeting mRNAs encoding receptors, kinases, scaffolding proteins, TFs, and regulatory modulators, mature miRNAs serve as powerful posttranscriptional regulators of signaling pathways [53]. miRNAs modulate pathway output by promoting translational repression or mRNA degradation through sequence-specific binding, mainly within the 3′UTR of target transcripts [54].

The combinatorial aspect of miRNA-mediated regulation—a single miRNA can simultaneously target many components across several signaling pathways—is one of its defining characteristics. By modifying signaling networks rather than discrete pathways, miRNAs can coordinate a variety of biological activities, including proliferation, survival, differentiation, and death [25].

In contrast, both canonical and noncanonical signaling pathways have a direct role in governing miRNA transcription, processing, and stability. The activation of TFs by pathways like Wnt/β-catenin, PI3K/AKT, NF-κB, and p53 controls miRNA gene promoters, connecting external signals to miRNA expression programs [55]. In addition to transcriptional regulation, signaling effectors can influence elements of the miRNA biogenesis machinery, such as DROSHA, DICER, and related RNA-binding proteins, affecting the availability and function of miRNAs [15].

These interactive relationships position miRNAs as not only downstream participants in signaling pathways but also as regulatory hubs that relay information back into upstream signaling networks. Consequently, the interplay between miRNAs and signaling pathways functions as a dynamic regulatory system rather than a simple one-way signaling hierarchy [52].

### 3.2. Context-Dependent Roles of Some miRNAs as Tumor Suppressors or OncomiRs

Some miRNAs exhibit context-dependent (dual) roles either as tumor suppressors or oncomiRs depending on the cellular lineage, target gene expression, signaling network architecture, disease progression, and the TME [25,56]. This functional duality arises because a single miRNA can bind and regulate multiple mRNAs depending on complementary target sequences, cell type, and cellular status. In addition, the functional duality of these miRNAs also arises due to the availability and expression levels of target genes, complexity of miRNA-mRNA interactions, competing endogenous RNA network, and dynamic changes in target interactions under cellular conditions [25].

In addition, a single mRNA can have binding sites for several different miRNAs allowing them to regulate its expression [57]. Thus, diverse signaling pathways involved in cell proliferation, differentiation, invasion, metastasis, and apoptosis are influenced by this duality [6]. Furthermore, miRNAs can regulate extrinsic variables that alter tumors, including interactions between the immune system and cancer, stromal cells, oncoviruses, and treatment sensitivity. In the end, whether a particular miRNA has a net oncogenic or net tumor-suppressive effect depends on how these mechanisms are balanced [58].

miR-21 primarily functions as an oncomiR and is overexpressed in several human cancers such as HCC, head and neck carcinoma, glioblastoma, ovarian cancer, B-cell lymphoma, and lung carcinoma [59,60]. Nonetheless, in contrast to such well-documented tumorigenic effects of miR-21, it is also reported that this miRNA elicits an antitumor immune response, thus revealing a dual function of miR-21 [61].

miR-155 exhibits an oncogenic role in hematologic malignancies and some solid tumors [62]. An aggressive lymphoma was reported in miR-155 Cre-loxP tetracycline-controlled knock-in mouse models by the overexpression of this miRNA [62]. However, in spite of its powerful oncogenic effects, it has also been reported that miR-155 possesses a tumor-suppressive function in certain cancers. It has been described that miR-155 restricts proliferation in various melanoma cell lines, partly by downregulating SKI (Sloan-Kettering Institute), an oncogene frequently overexpressed in melanoma [63]. Furthermore, the tumor-suppressive role of miR-155 has been reported in gastric cancer and ovarian cancer–initiating cells by targeting SMAD2 and CLDN1, respectively [64].

Dysregulated miR-200 expression in different human cancers links it to both tumor-promoting and tumor-suppressive roles [65]. The miR-200 family members, whether separately or together, have been documented to play roles in the control of EMT and metastasis [66], and the cell cycle and apoptosis [67]. Conversely, it has been confirmed that miR-200 effectively inhibit EMT across various cancer types by directly affecting the key EMT transcription factors ZEB1 and ZEB2 [68,69]. In addition, numerous studies have shown that members of the miR-200 family affect cancer cell invasion by modulating proteins that are key to cytoskeleton remodeling. This contradictory role of the miR-200 family highlights that these molecules perform roles influenced by the cellular environment and signaling pathways.

### 3.3. Feedback and Feed-Forward Loops as Determinants of Network Robustness

In reciprocal feedback loops (RFLs), two genes engage in mutual regulation, which is frequently employed to create bidirectional control and alternate between different cellular states [70]. Often, these feedback loops consist of a miRNA and a TF, and when the feedback is mutually repressive, they can drive a substitute cellular state. A relevant example in neuroblastoma (NB) is the mutually repressive feedback between miR-34a and MYC proto-oncogene, N-Myc (MYCN) [71,72], as well as the interaction between the miR-200 family and the ZEB TFs [73,74], which regulate the phenotypic switch during EMT that occurs prior to cell movement in various developmental processes, including neural crest cell (NCC) migration.

The participating TF can possibly trigger the expression of a miRNA involved in its repression, thereby supporting homeostasis by effectively regulating its own expression when it surpasses a specific threshold. In midbrain dopamine neurons, pertinent to neurogenesis, Pitx3, working as a TF, activates a series of genes essential for the maturation of dopamine neurons, while also stimulating the transcription of miR-133b, which posttranscriptionally inhibits paired-like homeodomain TF3 (Pitx3) [75].

Feed-forward loops (FFLs) consist of three elements: miRNA, TF, and a target gene. In this configuration, the TF governs both the miRNA and the target gene, while the miRNA also directly influences the target gene. FFLs can be classified as either coherent or incoherent, based on the regulatory outcomes. In coherent FFLs, the miRNA and TF support each other’s functions, leading to stable and decisive gene expression [76,77]. For instance, miR-124 facilitates neurogenesis by directly targeting genes associated with the cell cycle and by inhibiting E74-like factor 4 (ELF4), which otherwise encourages an undifferentiated condition by increasing the expression of several of these same cell-cycle genes [78].

The activation of miR-17~92 transcription by the proto-oncogene MYCN forms a coherent FFL, in which the miR-17~92 cluster and its associated members target genes responsible for apoptosis and cell-cycle blockade. This mechanism amplifies the MYCN-initiated proliferative and survival signals [79]. By contrast, incoherent FFLs involve TFs and miRNAs that exert opposing influences on a target gene. A good example of this phenomenon involves the miR-34a and NUMB (a miR-34a target)-mediated mutual repression of NOTCH. gene-expression noise is buffered and Notch expression is enhanced, which is significant in cancer stem cells [80].

A mechanistic understanding of miRNA-signaling pathway crosstalk requires distinguishing experimentally validated miRNA-mRNA interactions from computational predictions. Direct targeting is commonly assessed using wild-type and mutant 3′UTR luciferase reporter assays, complemented by miRNA mimic/inhibitor experiments, target-expression analysis, and functional rescue studies [25]. Argonaute-associated RNA immunoprecipitation and crosslinking-based methods provide additional evidence of miRNA-target interactions; however, binding alone does not establish functional repression [81,82].

## 4. Dysregulated miRNA-Signaling Axis in Core Oncogenic Networks

### 4.1. PI3K/AKT/mTOR Pathway

**Figure 2:**
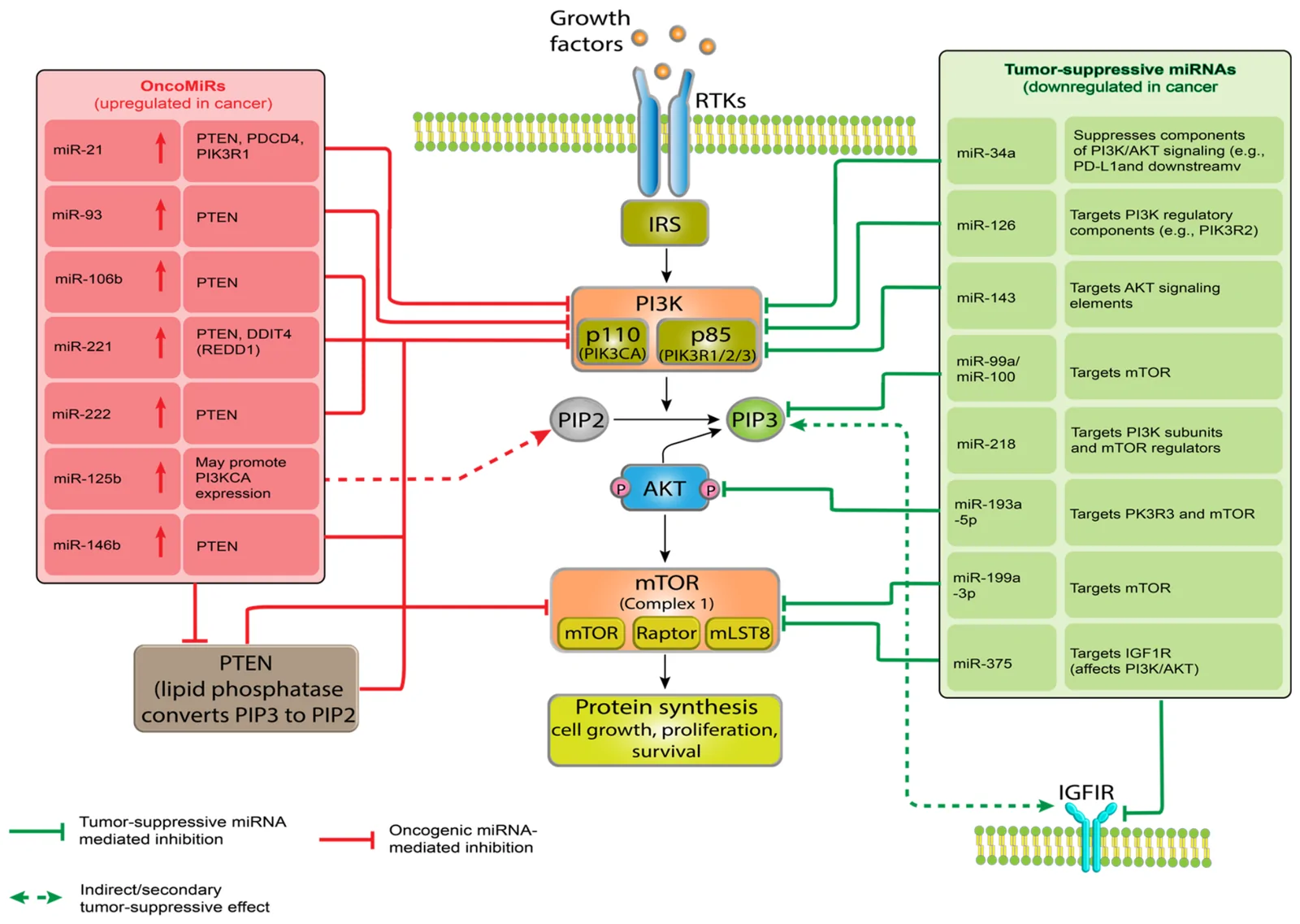
PI3K/AKT/mTOR signaling pathway and the role of some tumor-suppressive and oncogenic miRNAs, targeting important key components of this pathway. Abbreviations: AKT, Protein Kinase B (PKB); DDIT4, DNA Damage-Inducible Transcript 4; IGFIR, Insulin-like Growth Factor 1 Receptor; IRS, Insulin Receptor Substrate; mLST8, Mammalian Lethal with SEC13 Protein 8 (also known as GβL); mTOR, Mechanistic Target of Rapamycin; PDCD4, Programmed Cell Death 4; PIK3R1, Phosphoinositide 3-Kinase Regulatory Subunit 1; PI3K, Phosphoinositide 3-Kinase; PIK3CA, Phosphatidylinositol-4,5-bisphosphate 3-Kinase Catalytic Subunit Alpha; PIK3R1/R2, Phosphoinositide 3-Kinase Regulatory Subunits 1 and 2; PIP2, Phosphatidylinositol 4,5-Bisphosphate; PIP3, Phosphatidylinositol 3,4,5-Trisphosphate; PTEN, Phosphatase and Tensin Homolog; REDD1, Regulated in Development and DNA Damage Responses 1; RTKs, Receptor Tyrosine Kinases.

The vital processes of a cell like its survival, metabolism, growth, and angiogenesis are controlled by the PI3K/AKT/mTOR pathway [83] and this pathway gets very frequently dysregulated in cancer (Figure 2). Some tumor-suppressive and oncogenic miRNAs in addition to other noncoding RNAs tightly regulate this pathway by modulating some key nodes [84]. Multiple tumor types have a strong association with stemness maintenance, therapy resistance, and immune evasion, which occur due to the dysregulation of the PI3K/AKT/mTOR pathway. Some examples are cell survival in prostate cancer, inhibition of apoptosis, and EMT. In this cancer, miR-101-3p is poorly expressed and if its expression is restored by blocking the PI3K/AKT/mTOR pathway, the cancer cells can suppress migration and restore apoptosis [85].

Some cell lines like LNCaP and DU145 have revealed that phosphatidylinositol-4,5-bisphosphate 3-kinase catalytic subunit alpha (PIK3CA), present in these cells, is directly targeted by miRNA-101-3p. This targeting reduces downstream phosphorylation of AKT and the expression of EMT markers. Similarly, in pancreatic ductal adenocarcinoma (PDAC), miR-194-5p regulates the poliovirus receptor-related protein 3 (PVRL3)/miR-194-5p/SOCS2 axis and modulates proliferation and metastasis. The upregulation of this axis activates the PI3K/AKT/mTOR pathway. Luciferase reporter, RNA immunoprecipitation, and rescue assays validated the circPVRL3/miR-194-5p/SOCS2 axis and demonstrated that miR-194-5p-mediated SOCS2 suppression activates PI3K/AKT signaling and promotes PDAC proliferation and metastasis [86]. Although SOCS2 primarily regulates JAK/STAT signaling, its suppression by miR-194-5p was associated with PI3K/AKT activation in PDAC, suggesting pathway crosstalk [86]. These reports suggest that in PDAC, miR-194-5p functions as an oncogenic regulator, in contrast to miR-101-3p, which behaves as a tumor-suppressive marker. These examples emphasize the importance of different miRNAs that act on both upstream and downstream nodes of the PI3K/AKT/mTOR pathway, although a thorough understanding is needed.

Some miRNAs interconnect this pathway with other signaling pathways like JAK/STAT3 and Wnt/β-catenin and these pathways further share some effectors such as glycogen synthase kinase 3 beta (GSK3β). Cellular metabolic adaptation and proliferation are likewise regulated by these multidimensional and interconnected signaling pathways. The role of some tumor-suppressive and oncogenic miRNAs targeting key components of the PI3K/AKT/mTOR signaling pathway is illustrated in Figure 2 and Table 1.

**Table 1:** Role of some tumor-suppressive and oncogenic miRNAs targeting key components of the PI3K/AKT/mTOR signaling pathway and their functional implications.

miRNA	Primary Target(s) in PI3K/AKT/mTOR Pathway	Cancer Context	References
Tumor-suppressive miRNAs			
miR-34a	Suppresses components of PI3K/AKT signaling (e.g., PD-L1 and downstream)	Downregulates PI3K/AKT signaling and supports apoptosis/antitumor effects in breast, lung, and other cancers	[87]
miR-126	Targets PI3K regulatory components (e.g., PIK3R2)	Suppresses PI3K/AKT signaling and tumor growth in several cancers	[88]
miR-143	Targets AKT signaling elements	Reduces AKT pathway activity and inhibits cell proliferation	[88]
miR-99a/miR-100	Targets mTOR	Downregulation associated with enhanced mTOR signaling	[89]
miR-218	Targets PI3K subunits and mTOR regulators	Suppresses PI3K/AKT/mTOR signaling, reducing invasion and migration	[89]
miR-193a-5p	Targets PIK3R3 and mTOR	Lower expression linked to metastasis in non-small cell lung cancer (NSCLC)	[90]
miR-199a-3p	mTOR	Downregulated in cancers with activated mTOR signaling	[91]
miR-375	IGF1R (affects PI3K/AKT)	Acts as tumor suppressor and inhibits PI3K/AKT signaling in HCC and esophageal cancer	[92]
Oncogenic miRNAs			
miR-21	PTEN, PDCD4, PIK3R1	Overexpressed in many cancers; suppresses PTEN → PI3K/AKT/mTOR activation	[93]
miR-93	PTEN	Promotes PI3K/AKT activation in breast cancer	[94]
miR-106b	PTEN	Enhances PI3K/AKT signaling by PTEN suppression	[95]
miR-221	Targets DDIT4 (affects mTOR); PTEN	Enhances mTOR signaling in several cancers	[89]
miR-222	PTEN	Enhances PI3K/AKT signaling in cancers such as endometrial cancer	[96]
miR-125b	May promote PI3KCA expression	Elevated expression can contribute to PI3K/AKT/mTOR activation in select cancers	[89]
miR-146b	PTEN	Promotes PI3K/AKT hyperactivation in thyroid cancer	[97]

### 4.2. MAPK/RAS/ERK Signaling

**Figure 3:**
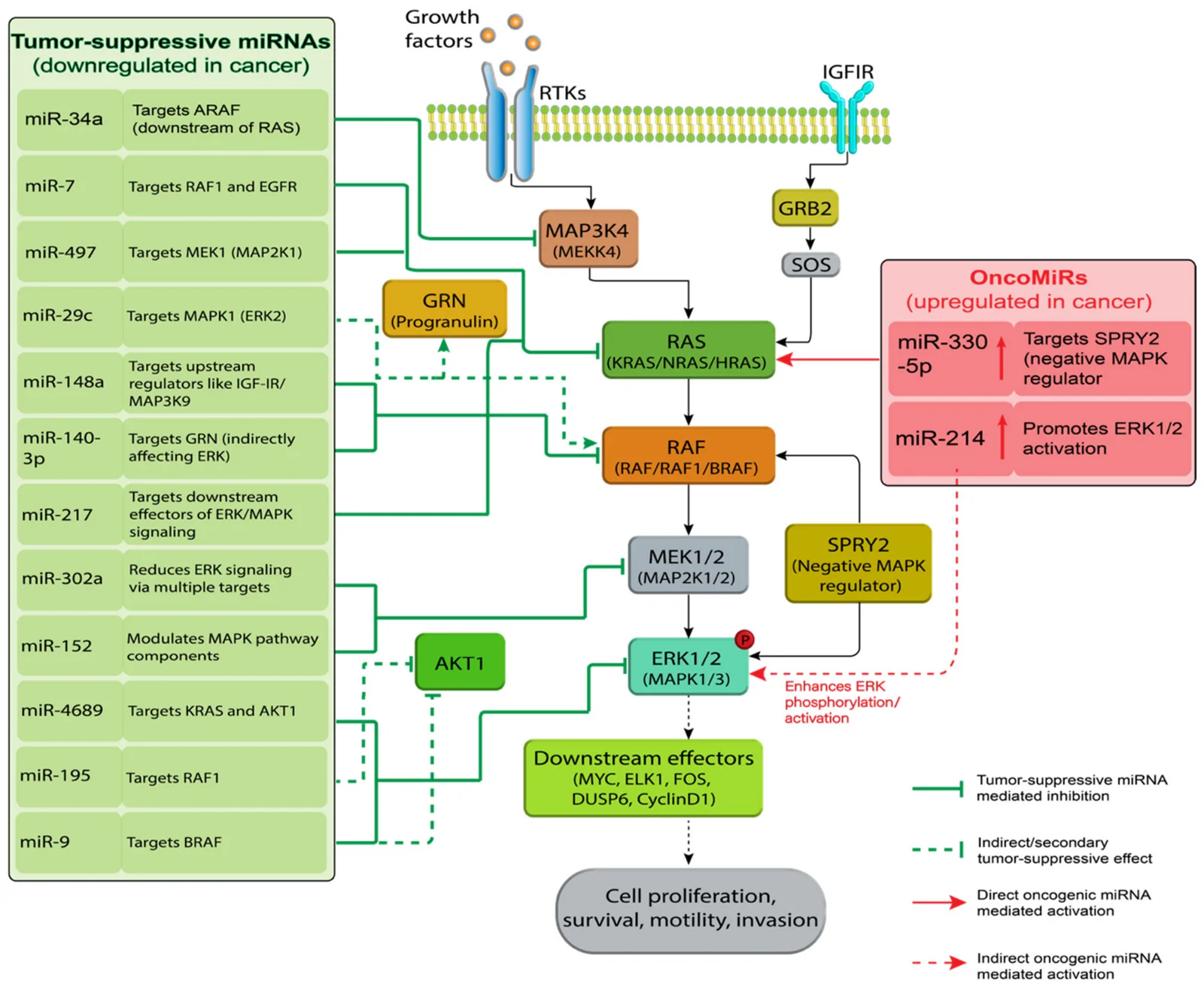
Role of some tumor-suppressive and oncogenic miRNAs targeting key components of the MAPK/ERK/RAS signaling pathway. Abbreviations: AKT1, AKT Serine/Threonine Kinase 1 (Protein Kinase B Alpha); DUSP6, Dual Specificity Phosphatase 6; ELK1, ETS Like-1; ERK1/2, Extracellular Signal-Regulated Kinases 1 and 2; FOS, FBJ Murine Osteosarcoma Viral Oncogene Homolog (Fos Proto-Oncogene); GRB2, Growth Factor Receptor-Bound Protein 2; GRN, Granulin; MAP3K4, Mitogen-Activated Protein Kinase Kinase Kinase 4; MEKK4, Mitogen-Activated Protein Kinase/ERK Kinase Kinase 4; MEK1/2, Mitogen-Activated Protein Kinase Kinases 1 and 2 (MAP2K1/2); MYC, MYC Proto-Oncogene, BHLH Transcription Factor; RAF, Rapidly Accelerated Fibrosarcoma; RAS, Rat Sarcoma; RTK, Receptor Tyrosine Kinase; SOS, Son of Sevenless; SPRY2, Sprouty RTK Signaling Antagonist 2.

The MAPK/RAS/ERK signaling pathway is well known to play a significant role in diverse cellular activities such as cell survival, growth, proliferation, migration, apoptosis, and differentiation [98] (Figure 3). This pathway transmits extracellular signals initiated by growth factors, cytokines, and mitogens through a kinase cascade involving RAS, RAF, MEK, and ERK [99]. Under normal conditions, this pathway is tightly controlled, but its dysregulation during cancer progression is a major driver of oncogenesis [100].

Tumor-suppressive miRNAs, such as miR-143, miR-145, and miR-34a, typically repress RAS family members, RAF kinases, or downstream effectors, thereby limiting ERK activation and controlling cell proliferation [101]. Loss or downregulation of these miRNAs is frequently observed in colorectal, pancreatic, lung, and other cancers, resulting in persistent RAS/ERK signaling that drives uncontrolled growth [17,102]. Conversely, oncomiRs such as miR-21 and miR-221/222 enhance ERK pathway activity by targeting negative regulators, including dual-specificity phosphatases (DUSPs) and Sprouty proteins, creating self-amplifying feedback loops that reinforce oncogenic signaling [103].

Experimental studies have confirmed miRNA-mediated regulation of oncogenic MAPK signaling. It has been validated that KRAS acts as a direct miR-143 target, demonstrating reduced ERK1/2 phosphorylation and colorectal cancer (CRC) cell proliferation using 3′UTR luciferase assays [104]. Furthermore, it has been reported that miR-34a targets genes involved in growth-factor signaling, with reduced ERK and AKT phosphorylation observed following miR-34a restoration. These results were observed by biotinylated miRNA pull-down assay, mutant 3′UTR reporter assays, and protein analysis. These findings link experimentally validated miRNA-target interactions to cancer-associated signaling dysregulation [105].

Different toxic substances and ionizing radiation can induce this pathway [106]. Some miRNAs including miR-34a, miR-193b, miR-181d, and miR-7-3 are closely associated with this pathway. Activation of the MAPK upregulates the expression of miR-7-3, while the expression of miR-34a, miR-193b, and miR-181d is downregulated. The role of some tumor-suppressive and oncogenic miRNAs targeting key components of the MAPK/RAS/ERK signaling pathway is illustrated in Figure 3 and Table 2.

Cancer cell proliferation is inhibited by the overexpression of MAPK-associated miRNAs, among which miR-193b plays a leading role. Along with the activation of upstream MAPK activity, the tumor-suppressive miRNAs inhibit the expression of multiple downstream targets, and this forms a complicated regulatory network [107]. miR-17-5p, usually overexpressed in the cancerous state by acting as an oncogene, effectively activates the p38 MAPK pathway by enhancing the phosphorylation of heat shock protein 27 (HSP27) [108].

The phosphatase and tensin homolog (PTEN) gene can be directly targeted by miR-21, leading to the upregulation of AKT and ERK 1/2 signaling pathways. This promotes the expression of vascular endothelial growth factor (VEGF) and hypoxia-inducible factor-1α (HIF-1α), inducing tumor angiogenesis, promoting further tumor growth and metastasis [109]. Beyond linear regulation, some miRNAs are intimately involved in reciprocal crosstalk with MAPK/RAS/ERK signaling, as the pathway itself can modulate miRNA expression through downstream TFs like E26 Transformation-Specific 1 (ETS1) and c-MYC [110]. This bidirectional regulation generates complex feedback and feed-forward loops that maintain network robustness in normal cells but, when disrupted, contribute to sustained pathway hyperactivation in tumors [111].

Therapeutically, the miRNA-MAPK/RAS/ERK axis presents unique opportunities, as restoring tumor-suppressive miRNAs or inhibiting oncomiRs can simultaneously modulate multiple nodes within the network, potentially overcoming compensatory feedback mechanisms that limit the efficacy of conventional kinase inhibitors [112]. Understanding the dysregulated crosstalk between miRNAs and the MAPK/RAS/ERK pathway therefore provides critical mechanistic insight into tumor progression and identifies novel avenues for precision-targeted interventions in diverse cancers [113].

**Table 2:** Tumor-suppressive and oncogenic miRNAs targeting key components of the MAPK/RAS/ERK signaling pathway and their functional implications.

miRNA	Primary Target(s) in MAPK/RAS/ERK Pathway	Cancer Context	References
Tumor-suppressive miRNAs			
miR-34a	Targets ARAF (downstream of RAS)	Downregulates RAS/MAPK signaling; reduced expression in many cancers	[114]
miR-7	Targets RAF1 and EGFR	Inhibits MAPK/ERK activation; studied in lung, breast, GBM	[115]
miR-497	Targets MEK1 (MAP2K1)	Suppresses MEK1 and downstream ERK activation	[114]
miR-29c	Targets MAPK1 (ERK2)	Inhibits ERK/MAPK activation; suppresses pancreatic cancer tumorigenesis	[116]
miR-148a	Targets upstream regulators like IGF-IR/MAP3K9	Suppresses MAPK/ERK signaling and EMT in various cancers	[117]
miR-140-3p	Targets GRN (indirectly affecting ERK)	Suppresses MAPK signaling, EMT, invasion in HCC	[117]
miR-217	Downstream effectors of ERK/MAPK signaling	Reduces ERK pathway activity in colorectal cancer	[118]
miR-302a	Reduces ERK signaling via multiple targets	Dampens MAPK cascade in colorectal cancer models	[119]
miR-152	Modulates MAPK pathway components	Suppresses proliferation via MAPK inhibition in CRC	[117]
miR-4689	Targets KRAS and AKT1	Suppresses EGFR/RAS/MAPK signaling in CRC	[120]
miR-195	Targets RAF1	Downregulated in thyroid cancer; decreases RAF1/ERK signaling	[121]
miR-9	Targets BRAF	Reduces MAPK pathway activity and induces apoptosis in thyroid cancer	[121]
Oncogenic miRNAs			
miR-330-5p	Targets SPRY2 (negative MAPK regulator)	Increases MAPK/ERK activity in HCC	[117]
miR-214	Promotes p-ERK/ERK activation	Enhances MAPK/ERK signaling and proliferation	[117]

### 4.3. Wnt/β-Catenin Pathway

**Figure 4:**
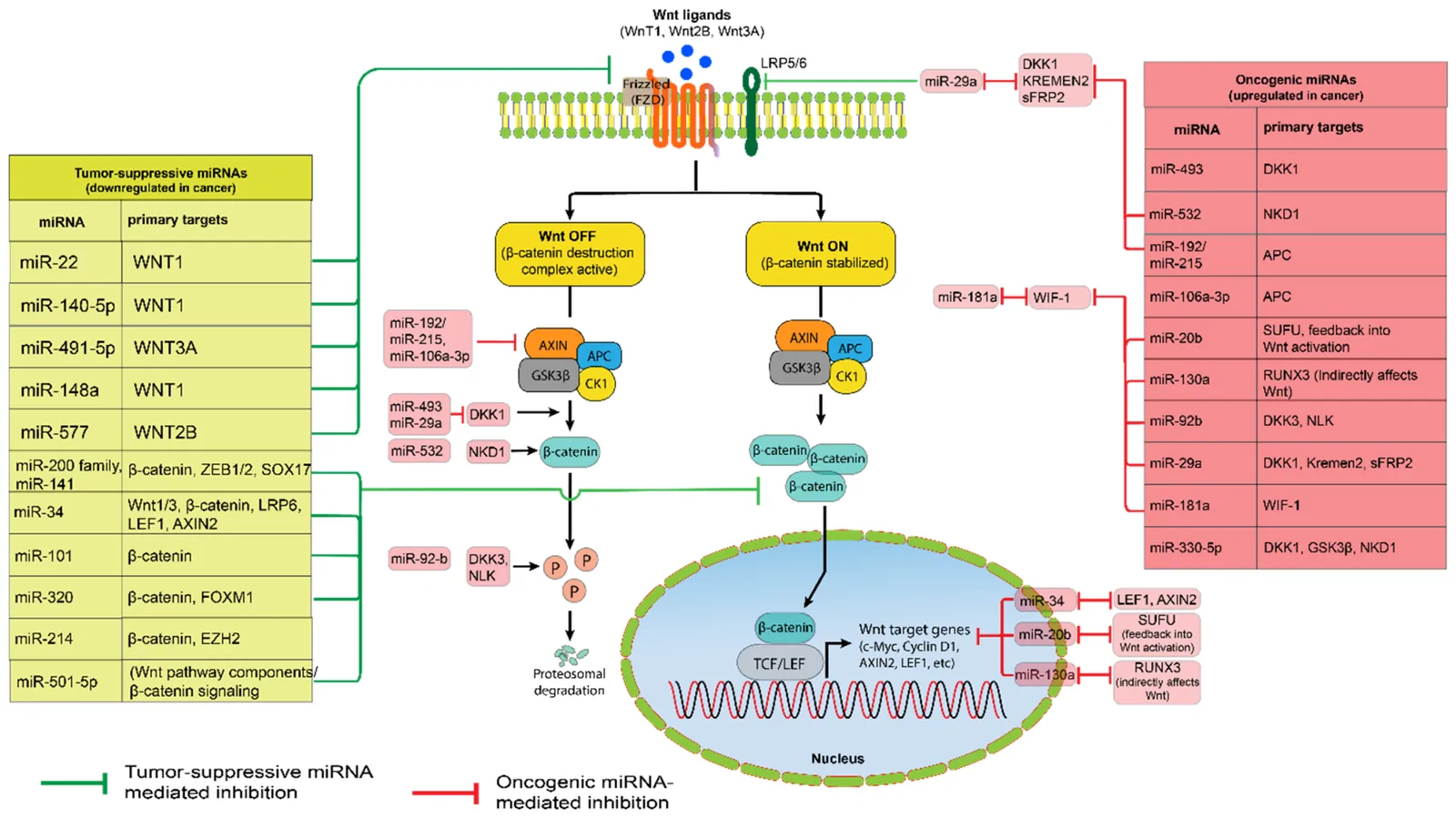
Role of some tumor-suppressive and oncogenic miRNAs targeting key components of the Wnt/β-catenin signaling pathway. Abbreviations: AXIN2, Axin 2; CK1, Casein Kinase 1; DKK1, Dickkopf WNT Signaling Pathway Inhibitor 1; FOXM1, Forkhead Box M1; EZH2, Enhancer of Zeste Homolog 2; KREMEN2, Kringle Containing Transmembrane Protein 2; LEF1, Lymphoid Enhancer-Binding Factor 1; LRP5/6, Low-Density Lipoprotein Receptor-Related Proteins 5 and 6; NKD1, Naked Cuticle Homolog 1; NLK, Nemo-Like Kinase; P, Phosphate/Phosphorylation; RUNX3, Runt-Related Transcription Factor 3; SOX17, SRY-Box Transcription Factor 17; SUFU, Suppressor of Fused; Wnt, Wingless-Related Integration Site; ZEB1/2, Zinc Finger E-Box-Binding Homeobox 1 and 2.

The Wnt/β-catenin signaling pathway regulates embryonic development, growth, and tissue homeostasis, maintenance of stem cells, and cellular differentiation [122], and the Wnt activation leads to the expression of target genes through β-catenin (Figure 4). The aberrant activation of this pathway is a hallmark of many cancers, including colorectal, hepatocellular, breast, and gastric malignancies [123]. The pathway is tightly controlled under normal conditions by the continued activity of the destruction complex, targeting β-catenin for ubiquitin-mediated degradation, preventing its nuclear accumulation and transcriptional activation of Wnt target genes [124].

Tumor-suppressive miRNAs such as the miR-200 family and miR-34a directly target β-catenin, Wnt ligands, or transcriptional coactivators such as T-cell factor (TCF)/lymphoid enhancer-binding factor (LEF), thereby restricting nuclear β-catenin accumulation and limiting the transcription of genes involved in proliferation, survival, and EMT [125]. Biochemical studies have validated miRNA-mediated regulation of Wnt/β-catenin signaling. miR-200a directly targeted the β-catenin (CTNNB1) 3′UTR, as demonstrated by luciferase reporter assays, while Western blotting, TOPFlash/FOPFlash assays, and cell migration and proliferation assays linked its expression to reduced Wnt signaling and tumor aggressiveness [126,127].

Conversely, oncogenic miRNAs, including miR-21, miR-135b, and miR-372/373, repress negative regulators of Wnt signaling, such as adenomatous polyposis coli (APC), glycogen synthase kinase 3β (GSK3β), and dickkopf-related protein 1 (DKK1), resulting in sustained β-catenin stabilization, nuclear translocation, and transcriptional activation of protumorigenic gene programs [128]. The role of some tumor-suppressive and oncogenic miRNAs targeting key components of the Wnt/β-catenin signaling pathway is illustrated in Figure 4 and Table 3.

Within the Wnt/β-catenin signaling pathway, depending upon the cellular context, some miRNAs perform dual roles by either promoting tumor progression or restoring homeostasis. It has been observed that the expression of miR-8063 was profoundly downregulated in colorectal cancer stem cells, whereas heterogeneous nuclear ribonucleoprotein A/B (hnRNP A/B) was markedly upregulated. In these types of cells, the hnRNP A/B overexpression promotes tumor- and colony-forming capabilities. However, restoration of miR-8063 expression downregulates the hnRNP A/B, Wnt3a, and β-catenin, diminishing the self-renewal of cancer stem cells, thus suppressing the tumorigenicity [129].

In contrast, it has been reported that in skin squamous cell carcinoma, expression of miR-664 is enhanced. It targets armadillo repeat containing 8 (ARMC8), which normally suppresses the Wnt/β-catenin pathway and EMT, leading to the migration and invasion of squamous cell carcinoma cells [130]. Thus, this miRNA relieves ARMC8-mediated repression of β-catenin signaling and functions as an oncogenic driver, promoting EMT and metastasis. In summary, these results emphasize how the Wnt/β-catenin signaling pathway is fine-tuned by some miRNAs, influencing the differentiation and plasticity of cancer stem cells, and EMT. Furthermore, an intertwined crosstalk between this pathway and other signaling pathways such as JAK/STAT3 and PI3K/AKT/mTOR, contributes to multidrug resistance and tumor recurrence.

**Table 3:** Role of some tumor-suppressive and oncogenic miRNAs targeting key components of the Wnt/β-catenin signaling pathway and their functional implications.

miRNA	Primary Target(s) in Wnt/β-Catenin Pathway	Cancer Context	References
Tumor-suppressive miRNAs			
miR-200a/miR-200b/miR-200c/miR-141 (miR-200 family)	β-catenin, ZEB1/2, SOX17	Suppresses Wnt/β-catenin signaling and EMT, inhibits metastasis in gastric cancer, HCC, breast cancer	[131]
miR-34	WNT1/3, β-catenin, LRP6, LEF1, AXIN2	Broad suppression of Wnt signaling, reduces downstream oncogenic transcription	[131]
miR-101	β-catenin	Reduces nuclear β-catenin accumulation; reciprocal inhibition with Wnt signaling	[132]
miR-320	β-catenin, FOXM1	Inhibits Wnt signaling and proliferation in prostate/colorectal cancer	[133]
miR-214	β-catenin, EZH2	Suppresses Wnt activity and oncogenic transcription	[132]
miR-22	WNT1	Inhibits Wnt ligand and pathway activation in gastric cancer	[134]
miR-140-5p	WNT1	Low expression correlates with increased Wnt signaling and gastric tumorigenesis	[135]
miR-491-5p	WNT3A	Inhibits Wnt3A and suppresses proliferation in gastric cancer	[134]
miR-148a	WNT1	Downregulated in NSCLC; inhibits Wnt signaling and metastasis	[136]
miR-577	WNT2B	Downregulation linked to increased Wnt/β-catenin signaling and NSCLC progression	[137]
Oncogenic miRNAs			
miR-501-5p	DKK1, GSK3β, NKD1	Promotes Wnt pathway activation and stem-like traits in gastric cancer	[134]
miR-493-3p	DKK1	Enhances proliferation, invasion, chemoresistance via Wnt activation	[138]
miR-532	NKD1	Promotes migration/invasion by activating Wnt signaling	[139]
miR-192/miR-215	APC	Reduces APC, activates Wnt/β-catenin, and drives proliferation in gastric cancer	[134]
miR-106a-3p	APC	Increases EMT/metastasis by downregulating APC	[140]
miR-20b	SUFU, feedback into Wnt activation	Promotes Wnt signaling and metastasis in gastric cancer	[141]
miR-130a	RUNX3 (indirectly affects Wnt)	Promotes Wnt signaling in HCC/gastric cancers	[131]
miR-92b	DKK3, NLK	Activates Wnt/β-catenin signaling, glioma progression	[142]
miR-29a	DKK1, Kremen2, sFRP2	Promotes resistance and Wnt activation in pancreatic cancer	[143]
miR-181a	WIF-1	Promotes Wnt signaling and tumor growth in colorectal cancer	[142]

### 4.4. TGF-β/SMAD and Other Developmental Pathways

**Figure 5:**
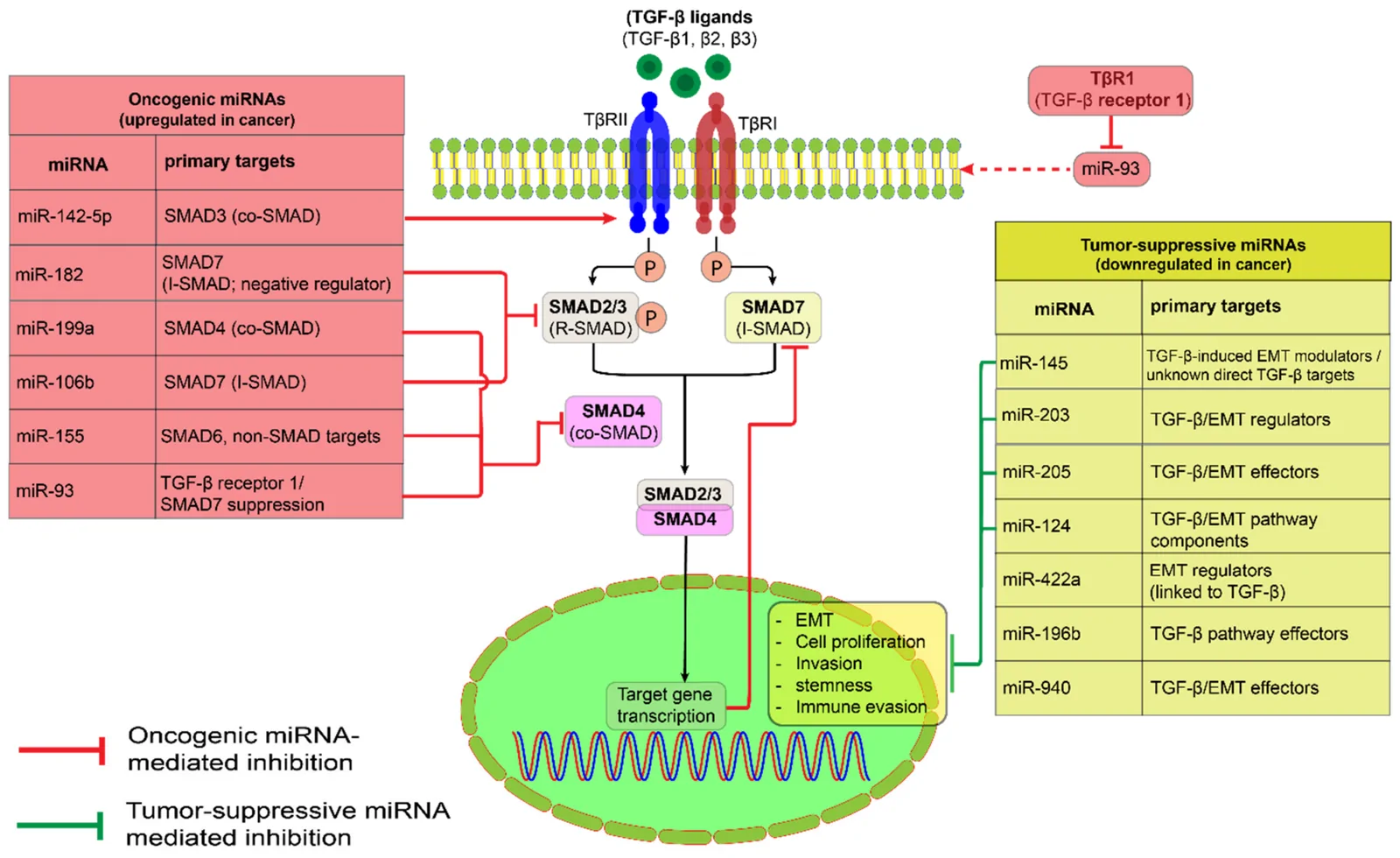
The role of some tumor-suppressive and oncogenic miRNAs targeting key components of the TGF-β/SMAD and other developmental signaling pathway. Abbreviations: co-SMAD; common-mediator SMAD; EMT, epithelial–mesenchymal transition; I-SMAD, Inhibitory SMAD; R-SMAD, receptor-regulated SMAD; TGF-β, transforming growth factor β; TβR1/II, transforming growth factor β receptor type I/type II; SMAD, SMAD proteins (Sma in *C. elegans* and Mad in *Drosophila*).

The transforming growth factor-β (TGF-β)/SMAD signaling pathway regulates cell growth, differentiation, and apoptosis. In normal cells, TGF-β ligands bind to type I and type II serine/threonine kinase receptors, initiating phosphorylation, leading to SMAD complex formation and translocation to the nucleus to regulate transcription of target genes [144] (Figure 5).

Some miRNAs can modulate this pathway; as reported in gastric cancer, miR-21-5p promotes stem-cell resistance to cisplatin by modulating the TGF-β/SMAD pathway [145]. TGF-β/SMAD signaling can also activate lncRNA plasmacytoma variant translocation 1 (PVT1) and promotes hepatocellular carcinoma through two distinct modes: the miR-143-3p/NRG1 axis and the miR-186-5p/SMAD6 axis [145]. The role of some tumor-suppressive and oncogenic miRNAs targeting key components of the TGF-β/SMAD signaling pathway is illustrated in Figure 5 and Table 4.

Some miRNAs critically regulate this TGF-β/SMAD axis by targeting ligands, receptors, and intracellular SMAD proteins, thereby fine-tuning pathway output and cellular responses [144]. Tumor-suppressive miRNAs, such as miR-34a and the miR-200 family, inhibit TGF-β-induced EMT and invasion by directly repressing SMAD2, SMAD3, or downstream TFs that mediate mesenchymal phenotypes. It has been reported that melanoma is inhibited by a tumor-suppressive miRNA such as miR-377. miR-377 was validated as targeting E2F3 and MAP3K7 (TAK1) in melanoma using 3′UTR luciferase reporter assays and binding-site mutagenesis, demonstrating reduced NF-κB signaling, proliferation, and migration [146]. Negative feedback is thus created, restraining chronic NF-κB activity and an inflammatory TME.

MAP3K7 acts as a core regulator of inflammation and cell proliferation, and is upregulated by the TGF-β/SMAD axis [147]. In addition to this signaling pathway activation, MAP3K7 also activates NF-κB, JNK, and p38, and these stimulate proliferative and prosurvival gene transcription [148,149]. It has been reported that TGF-β/SMAD intertwined with MAPK signaling promotes proliferation, EMT, and apoptosis not only through the mediation of miRNAs but also through circular RNAs (circRNAs) and long noncoding RNAs (lncRNAs). Thus, this crosstalk shows how miRNAs coordinate control over several oncogenic pathways.

**Table 4:** Role of some tumor-suppressive and oncogenic miRNAs targeting key components of the TGF-β/SMAD and other developmental signaling pathways and their functional implications.

miRNA	Primary Target(s) in TGF-β/SMAD Pathway	Cancer Context	References
Tumor-suppressive miRNAs			
miR-145	TGF-β-induced EMT modulators/unknown direct TGF-β targets	Suppresses EMT and metastasis; anti-oncogenic in NSCLC and other cancers	[137]
miR-203	TGF-β/EMT regulators	Inhibits EMT and limits metastatic spread associated with TGF-β signaling	[150]
miR-205	TGF-β/EMT effectors	Blocks TGF-β-induced EMT and reduces metastatic potential	[137]
miR-124-3p	TGF-β/EMT pathway components	Restores epithelial phenotype and inhibits metastatic progression	[151]
miR-422a	EMT regulators (linked to TGF-β)	Limits EMT and invasive behavior	[152]
miR-196b	TGF-β pathway effectors	Opposes EMT and tumor spread linked to TGF-β signaling	[137]
miR-940	TGF-β/EMT effectors	Reduces invasive phenotypes associated with TGF-β in NSCLC	[153]
Oncogenic miRNAs			
miR-142-5p	SMAD3 (co-SMAD)	Targets and suppresses SMAD3, attenuating TGF-β-mediated growth inhibition and promoting proliferation in cancer cells	[154]
miR-182	SMAD7 (I-SMAD; negative regulator)	TGF-β-induced miR-182 suppresses SMAD7, enhancing EMT and metastasis in breast and other cancers	[155]
miR-199a	SMAD4 (co-SMAD)	Represses SMAD4 expression, blocking the canonical TGF-β transcriptional response and promoting tumorigenesis (e.g., gastric cancer).	[156]
miR-106b	SMAD7 (I-SMAD)	Suppresses SMAD7, enhancing TGF-β/SMAD signaling and cancer-stem-cell traits in gastric cancer	[157]
miR-155	SMAD5/non-SMAD targets	Upregulated by TGF-β and contributes to EMT, migration, and invasion in breast cancer	[158]
miR-93	TGF-β receptor I/SMAD7 suppression	Promotes EMT and invasion via suppression of TGF-β negative regulators (NSCLC context)	[137]

### 4.5. JAK/STAT3 and Inflammatory Signaling Networks

**Figure 6:**
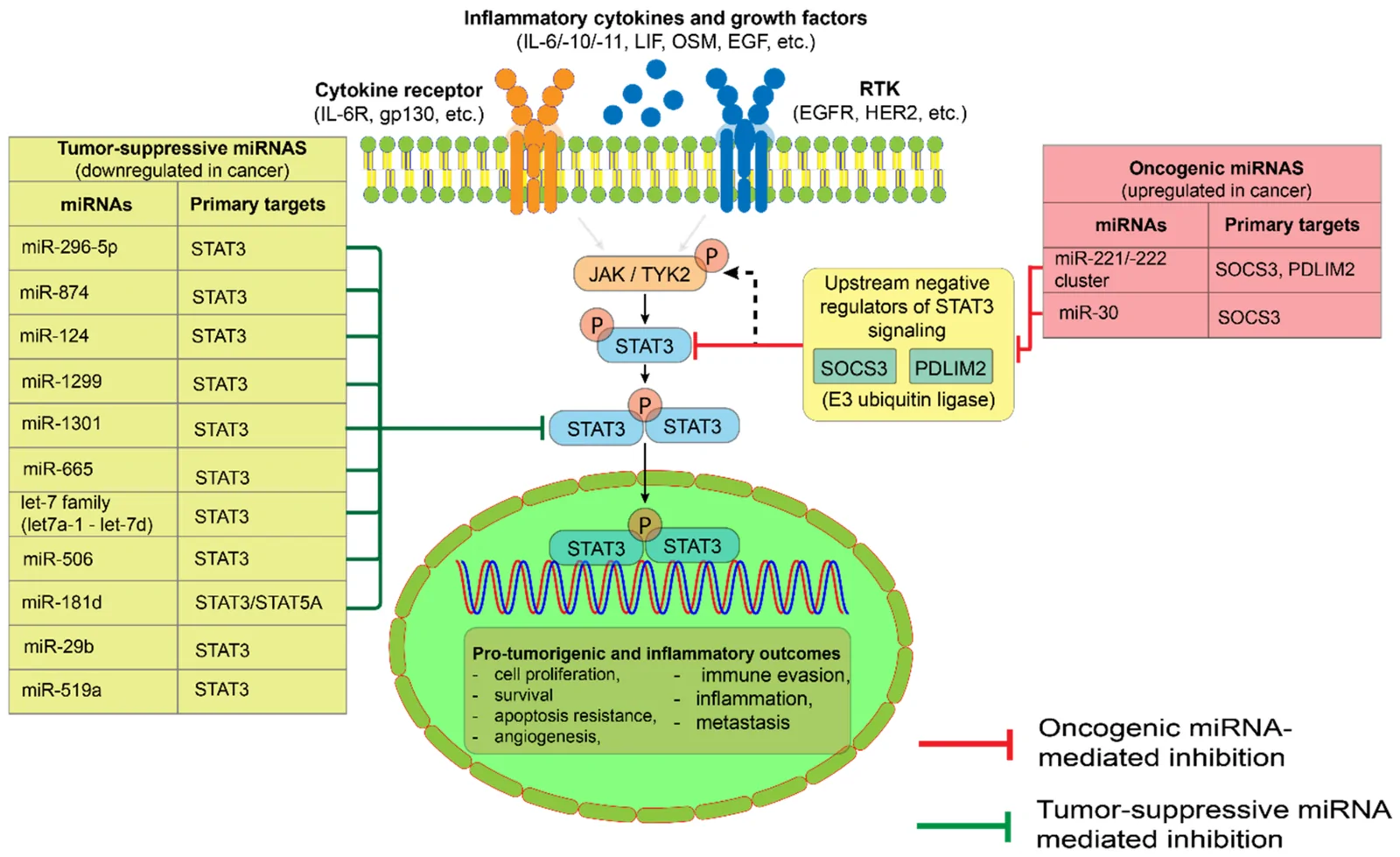
Role of different tumor-suppressive and oncogenic miRNAs targeting key components of the STAT3 and inflammatory signaling networks. Abbreviations: EGF, epidermal growth factor; EGFR, epidermal growth factor receptor; gp130, glycoprotein 130; HER2, human epidermal growth factor receptor 2; IL, interleukin; JAK, Janus kinase; LIF, leukemia inhibitory factor; OSM, oncostatin M; P, phosphate/phosphorylation; PDLIM2, PDZ and LIM domain protein 2; RTK, receptor tyrosine kinase; STAT, signal transducer and activator of transcription; SOCS3, suppressor of cytokine signaling 3; TYK2, tyrosine kinase 2.

The signal transducer and activator of transcription 3 (STAT3) signaling pathway is a central mediator of inflammatory and oncogenic responses in cancer, integrating signals from cytokines, growth factors, and stress-related stimuli [159] (Figure 6). This pathway regulates gene-expression programs that govern survival, proliferation, immune evasion, angiogenesis, and metastasis [159]. Under physiological conditions, STAT3 is transiently activated by receptor-associated Janus kinases (JAKs), which phosphorylate STAT3, leading to its dimerization, nuclear translocation, and transcriptional regulation of target genes [160].

The JAK/STAT3 cascade is vital to several biological processes such as cell cycle and proliferation [161,162]. Different oncogenic stimulants or cytokines activate STAT3, which is normally present in the cytoplasm, is translocated to the nucleus, and promotes gene transcription for survival, immune suppression, and proliferation. Thus, persistent activation of STAT3 leads to cancer progression [163,164]. The role of some tumor-suppressive and oncogenic miRNAs targeting key components of the JAK/STAT3 signaling pathway is illustrated in Figure 6 and Table 5.

Different miRNAs directly or indirectly participate in tumor progression by modulating JAK and STAT3 signal-transduction members. Different experimental evidence confirms that some miRNAs and antitumor drugs act by inhibiting JAK/STAT3 signaling. For example, as reported in lung cancer and osteosarcoma, miR-124 and miR-125 mimics synergize with STAT3 inhibitors and minimize the cancer progression [165,166].

In murine xenografts and pancreatic cancer cell lines (PANC-1 and BxPC-3), the overexpression of miR-301a inhibits SOCS5 and activates JAK/STAT3 signaling and leads to angiogenesis, cell proliferation, and migration [167]. It has been found that ovarian tumors synthesize extracellular vesicles (EVs). These EVs are rich in miR-141-3p and are engulfed by endothelial cells. These EVs release miR-141-3p, which minimizes the level of SOCS5, thus promoting angiogenesis by activating the JAK/STAT3 pathway [168]. These observations illustrate how exosomal miRNAs produced by tumors can remodel the vasculature for the support of growth and metastasis.

Glioma is initiated by glioma stem cells, which overexpress miR-30. This miRNA inhibits JAK/STAT3 signaling upon its downregulation, thus reducing cancer progression [169]. SOCS3 was reported to act as a direct miR-30 target using 3′UTR luciferase reporter assays with wild-type and mutant binding sites, supported by immunoblotting. The study further employed STAT3-responsive luciferase reporter assays, SOCS3 siRNA-mediated silencing, and in vivo tumorigenesis assays to investigate the functional role of the miR-30/SOCS3/JAK-STAT3 axis [169]. Similarly, it has been reported that in NSCLC cells, ubiquitin-specific peptidase 22 (USP22) is targeted by miR-30e-5p, which attenuates JAK/STAT3 signaling, suppressing these cells and cancer progression [170].

In NSCLC, miR-140-3p acts as a tumor-suppressive miRNA by directly targeting mineral dust-induced gene (MDIG), thereby attenuating STAT3 signaling, inhibiting cancer-cell proliferation, and promoting apoptosis [171]. Furthermore, liver cancer tissue and its cell lines are abundant in miR-500a-3p, and its overexpression upregulates tumorigenic markers and promotes cancer-stem-cell proliferation. It is reported that protein tyrosine phosphatase nonreceptor type 11 (PTPN11) in addition to SOCS2 and SOCS4 can negatively regulate JAK/STAT3 signaling, and these markers are repressed by miR-500a-3p [172]. When this pathway is inhibited by pharmacological means, it suppresses the expression of miR-500a-3p. Thus, a feedback mechanism is maintained, sustaining stem-cell traits. In contrast, activation of miR-500a-3p inhibitors restores SOCS function, limiting the capacity for self-renewal, thus emphasizing the potential of combined miRNA-drug therapies.

**Table 5:** Tumor-suppressive and oncogenic miRNAs targeting key components of the STAT3 and inflammatory signaling networks and their functional implications.

miRNA	Primary Target(s) in STAT3 Pathway	Cancer Context/Notes	References
Tumor-suppressive miRNAs			
miR-296-5p	STAT3	Directly targets STAT3, suppresses proliferation, migration, and invasion in colorectal and esophageal cancer	[173]
miR-874	STAT3	Downregulated in multiple cancers; directly suppresses STAT3 expression and tumor growth	[174]
miR-124	STAT3	Targets STAT3, inhibits cell proliferation, and induces apoptosis in esophageal and colorectal cancer	[173]
miR-1299	STAT3	Direct STAT3 targeting reduces proliferation and enhances apoptosis in colon cancer	[175]
miR-1301	STAT3	Downregulation promotes invasion and migration via enhanced STAT3 activation in CRC	[176]
miR-665	STAT3	Suppresses STAT3 signaling and colorectal cancer stem cell (CSC) stemness and proliferation	[173]
let-7 family (let-7a-1 ~ let-7d)	STAT3	Directly inhibits STAT3 and suppresses proliferation and survival in glioma	[177]
miR-506	STAT3	Inhibits STAT3, reduces proliferation/migration/invasion in glioma	[177]
miR-181d	STAT3/STAT5A	Suppresses migration and invasion via targeting STAT3/STAT5 in glioma	[178]
miR-29b-3p	STAT3	Inhibits STAT3 and chemoresistance in glioma	[179]
miR-519a	STAT3	Inhibits proliferation, invasion, and chemoresistance by targeting STAT3 in glioma	[177]
Oncogenic miRNAs			
miR-221/222 cluster	SOCS3/PDLIM2 (upstream negative regulators of STAT3)	Suppresses SOCS3 and PDLIM2, leading to increased STAT3 activation and tumor progression	[177]
miR-30	SOCS3	Increases p-STAT3 expression and promotes tumorigenesis in glioma	[169]

### 4.6. Notch Signaling Pathway

**Figure 7:**
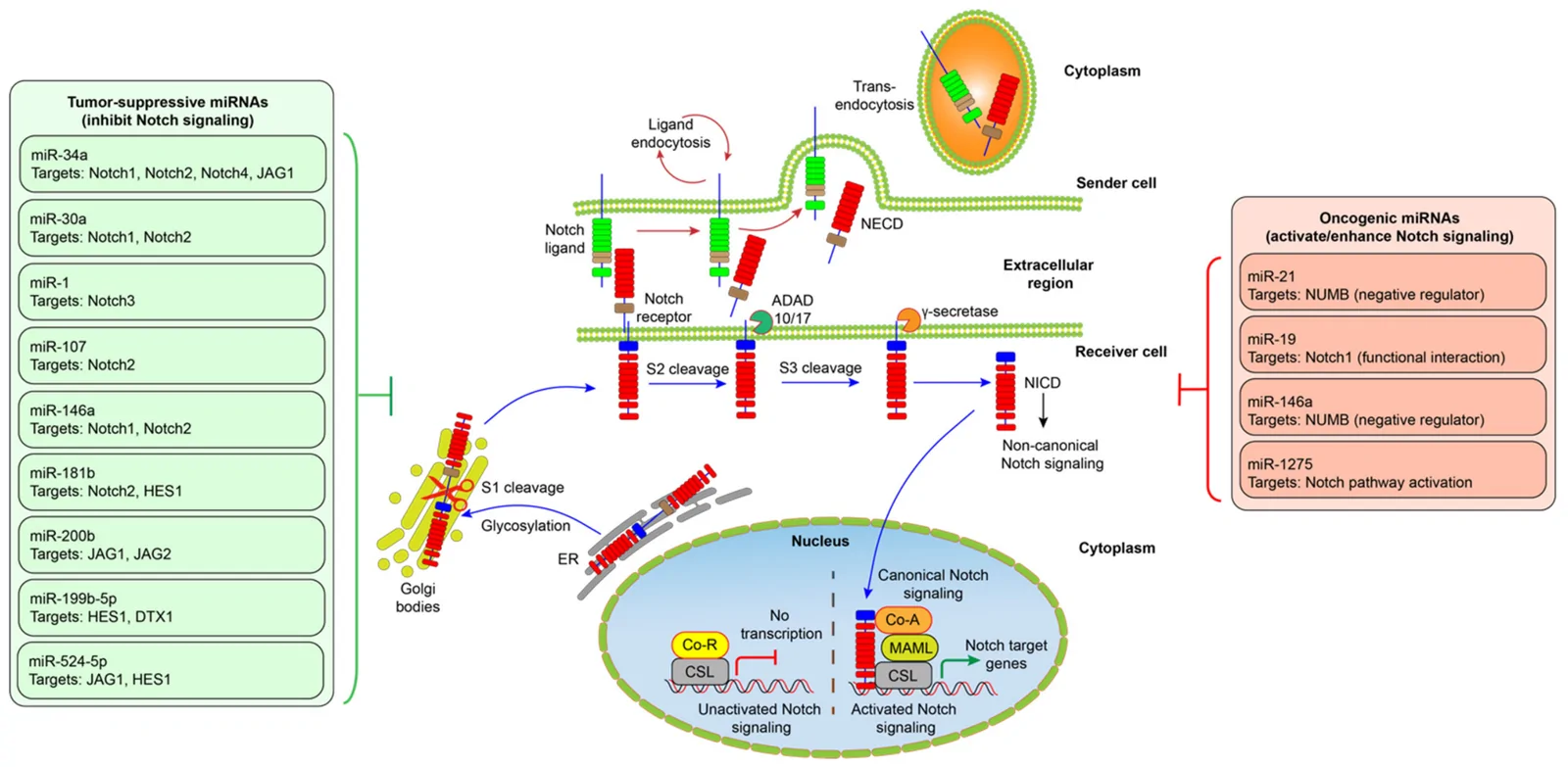
Role of different tumor-suppressive and oncogenic miRNAs targeting key components of the Notch signaling pathway. Abbreviations: ADAM10/17, A disintegrin and metalloproteinase 10/17; Co-A, coactivator; Co-R, corepressor; CSL, CBF1/Suppressor of hairless/LAG-1; DTX1, Deltex 1; ER, endoplasmic reticulum; HES1, Hes family BHLH transcription factor 1; JAG1, Jagged 1; MAML, Mastermind-like protein; NECD, Notch extracellular domain; NICD, Notch intracellular domain; NUMB, NUMB endocytic adaptor protein.

Notch signaling is a well-known pathway that regulates cell differentiation, proliferation, tissue homeostasis, and organ development [180]. This pathway is particularly significant in immune recognition, embryogenesis, stem-cell maintenance, and cancer biology [181]. The Notch receptor and its ligand are transmembrane proteins present on neighboring cells, and their interaction initiates the signal transduction (Figure 7). This pathway has a context-dependent dual role, serving as both oncogenic and tumor-suppressive signaling in different cancer types [180]. Dysregulation of this pathway leads to EMT and angiogenesis, closely related to proliferation, invasion, and metastasis [182].

The Notch signaling pathway is initiated when a membrane-bound ligand, such as Delta-like (DLL1 or DLL4) or Jagged (JAG1 or JAG2), present on neighboring cells, binds to a Notch receptor (Notch 1–4) present on the other cell [183]. This binding leads to a conformational change exposing the receptor to proteolytic cleavage by ADAM10 or ADAM17 (a disintegrin and metalloprotease 10/17). Furthermore, cleavage by γ-secretase complex promotes the release of Notch intracellular domain (NICD) [184]. This domain translocates into the nucleus and binds to the transcription factor RBPJ/CSL, recruiting mastermind-like (MAML) coactivators and forming a transcriptional activation complex. This complex induces the expression of downstream target genes depending on the cellular context and interactions with other signaling pathways [185]. The role of some tumor-suppressive and oncogenic miRNAs targeting key components of the Notch signaling pathway is illustrated in Figure 7 and Table 6.

The Notch signaling pathway is targeted by different miRNAs such as miR-19 [186], miR-21 [187], miR-146a [188], miR-100 [189], miR-181a-1/b-1 [190], and miR-375 [191], which promote cancer initiation and progression. A study showed that miR-146a expression enhances the proliferation of human melanoma cells in culture and promotes tumor formation in mice. However, the knockdown of this miRNA leads to opposite effects as miR-146a targets the NUMB mRNA (a Notch repressor) and leads to these oncogenic activities [188].

It was reported that as compared to normal tissues, CRC tissue samples showed overexpression of miR-21 and Notch1, presenting a positive correlation with tumor progression, especially in the advanced state [187]. This observation led to the conclusion that in CRC a crosstalk occurs between miR-21 and the Notch signaling pathway. In another study, it was observed that in stem cells, Notch2 and Hes1 are targeted by miR-34a, which suppresses this signaling pathway. As a result, the odontogenic and osteogenic differentiation is repressed by Notch signaling [192].

**Table 6:** Tumor-suppressive and oncogenic miRNAs targeting key components of the Notch signaling and their functional implications.

miRNA	Primary Targets in Notch Pathway	Cancer Context	References
Tumor-suppressive miRNAs			
miR-34a	Notch1, Notch2, JAG1	Cholangiocarcinoma	[193]
miR-30a-5p	Notch1, Notch2	Diffuse large B-cell lymphoma; T-cell acute lymphoblastic leukemia	[194]
miR-1	Notch3	CRC	[195]
miR-146a	Notch1	Glioma	[196]
miR-146a-5p	Notch2	Esophageal squamous cell carcinoma	[197]
miR-181b	Notch2; inhibits Notch2/HES1 signaling	NSCLC	[198]
miR-524-5p	JAG1 and HES1	Glioma	[199]
miR-199b-5p	Notch pathway components	Osteosarcoma	[200]
Oncogenic miRNAs			
miR-21	Notch1-associated signaling	CRC	[187]
miR-19	Notch1-associated regulatory network	T-cell acute lymphoblastic leukemia	[186]
miR-146a	NUMB (direct target), negative regulator of Notch signaling	Breast cancer models	[201]
miR-1275	NUMB (direct target), also regulates Wnt/β-catenin signaling	Lung adenocarcinoma	[202]

## 5. Integrated Network Dysregulation in Cancer Phenotypes

### 5.1. Chemoresistance

It is now well understood that cancer is a disease of dysregulated signaling networks rather than isolated gene mutations, and miRNAs are central mediators of this network-level regulation. miRNAs integrate multiple tumor-suppressive and oncogenic signals to determine cellular phenotypes [203]. Dysregulated miRNAs result in simultaneous perturbation of PI3K/AKT/mTOR, MAPK/RAS/ERK, Wnt/β-catenin, TGF-β/SMAD, and STAT3/inflammatory signaling, and create aberrant signaling that drives malignant behaviors [204].

It is reported that the Enhancer of Zeste Homolog 2 (EZH2)/STAT3 signaling pathway is activated by some chemotherapeutic agents such as paclitaxel and doxorubicin, and leads to enhanced secretion of exosomes containing miR-378a-3p and miR-378d. The breast cancer chemoresistant cells engulf these exosomes and intracellularly released miRNAs activate Notch and Wnt/β-catenin signaling pathways by inhibiting DKK3 (Wnt/β pathway inhibitor) and NUMB (Notch pathway inhibitor), conferring breast cancer-stem-cell characteristics with enhanced chemoresistance. Furthermore, the application of EZH2 inhibitors such as tazemetostat significantly decreases the level of miR-378a-3p and miR-378d in exosomes and restores the expression of NUMB and DKK3 and reverses the chemoresistance effects [205].

The plasma and breast cancer (BC) cell lines (MCF-7/ADR), obtained from chemotherapy-resistant patients showed significantly downregulated levels of miR-1275. This low-level expression of miR-1275 showed a strong correlation with reduced patient overall survival rate. Overexpression of miR-1275 was found to directly target the 3′UTR of MDK (midkine) and was responsible for the enhanced sensitivity of BC cells to chemotherapeutic agents like epirubicin. MDK, a secreted protein, activates PI3K/AKT signaling, thus imparting chemoresistance [206].

Downregulated expression of miR-508-5p, which acts as a tumor-suppressor microRNA in endometrial cancer (EC) cells, is linked with chemoresistance in EC stem cells. miRNA-508-5p targets the 3′UTR and inhibits SOX2 expression, subsequently minimizing the capability of ECSCs for self-renewal, chemoresistance, and invasion. Stem cells use SOX2 as a TF for their development and pluripotency. Thus, it is used as a principal oncogenic driver as it preserves CSC characteristics, activates oncogenic pathways, promotes EMT, and fosters treatment resistance [207,208].

In ovarian cancer, Wnt/β-catenin signaling is promoted by miR-181a, as this miRNA targets and inhibits SFRP4 (Wnt pathway inhibitor). Thus, the ovarian cancer-stem-cell trait is augmented by resisting platinum-based chemotherapy. Upregulated expression of ovarian cancer cell miR-181a is strongly correlated with therapeutic treatment resistance. Moreover, accumulated β-catenin and the upregulated Wnt expression target some genes including aldehyde dehydrogenase (ALDH), catenin β-1 (CTNNB1), cluster of differentiation 133 (CD133), lymphoid enhancer-binding factor 1 (LEF1), leucine-rich repeat containing G protein-coupled receptor 5 (LGR5), SRY-box transcription factor 2 (SOX2), and transcription factor 7 (TCF7). Furthermore, minimal expression of SFRP4 is associated with poor ovarian cancer prognosis [209].

### 5.2. EMT, TME, and Metastasis

miRNAs can either promote or inhibit EMT as more than 130 different miRNAs have been discovered to regulate the EMT process [210]. Different miRNA targets involved in EMT include TFs such as ZEB1/2, TWIST, and SNAIL. miR-200 family members, miR-141, and miR-429 target mRNAs of EMT-promoting factors and result in their inhibition. miR-200 overexpression promotes enhanced E-cadherin expression, involved in epithelial phenotype maintenance and EMT inhibition [211]. The E-cadherin expression is reduced by ZEB1/2 when targeted by miR-200 members [68,212]. The expression of ZEB1 and ZEB2 also downregulates TP53, which activates miR-200 and miR-192, which reciprocally repress ZEB1 and ZEB2, thus resulting in the inhibition of EMT [213].

Apart from the miR-200 family, miR-9, miR-10b in breast cancer cells inactivate E-cadherin expression and promote cell motility and metastasis [214,215]. It has also been reported that miR-29b and miR-30a-5p inhibit SNAIL1 expression [156,216]. miR-29b expression is downregulated in prostate cancer, and its expression downregulates mesenchymal markers and amplifies epithelial markers [217]. Furthermore, SNAIL1 and miR-34 both negatively control each other’s expression [218]. SNAIL1 downregulates the miR-34 expression during TGF-β-induced EMT.

As miRNA expression is dysregulated in most cancers, it affects the communication between tumor cells and the stroma. Thus, for fully understanding the molecular mechanisms of cancer progression and its therapeutic intervention, understanding the role of miRNAs in the TME is equally important. The role, significance, and number of miRNAs participating in the TME are expanding day by day [219,220]. It is reported that miRNAs have a vast role in the TME as some miRNAs regulate the expression of cancer-associated fibroblasts (CAFs), cancer-associated mesenchymal stem cells (MSCs), tumor-associated macrophages (TAMs), T cells, and endothelial cells [221].

It was reported that the ablation of the PTEN gene leads to a significant decrease in the expression of miR-320, and this induces the conversion of normal fibroblasts in BC to CAFs [222]. Similarly, miR-9 induced the conversion of normal fibroblasts to CAFs in BC [223]. Furthermore, miR-200 downregulation also reprogrammed the conversion of normal fibroblasts into CAFs. miR-200 targets transcription factor 12 and friend leukemia virus integration 1 (Fli-1) in BCs [224,225].

It has been demonstrated that downregulation of miR-155-5p induces bone marrow mesenchymal stem cells (MSCs) to acquire the gastric cancer MSC phenotype by NF-κB p65 activation. NF-κB kinase inhibitor and NF-κB p65 have been found to be targeted by miR-155-5p [226]. It has further been reported that miR-222, miR-214, and miR-155-5p are also upregulated in gastric cancer MSCs. Gastric cancer MSCs considerably promote migration and proliferation of gastric cancer cells. In gastric cancer MSCs, the targeted inhibition of miR-221 inhibits cancer progression effects [227].

miRNAs significantly participate in different steps of metastasis. In a mouse xenograft model, it was reported that miR-126/miR-126* independently inhibited the recruitment of inflammatory monocytes and MSCs to the tumor stroma, suppressing lung metastasis by the downregulation of C-C motif chemokine ligand 2 (CCL2) and stromal cell-derived factor-1α [228]. Cancer cell- and fibroblast-secreted miR-9 stimulates migration of BC cells by reducing E-cadherin [223]. It has been reported recently that miR-200 from CAFs enhances the expression of Fli-1 and TCF12 and promotes stromal ECM remodeling and drives metastasis by upregulating lysyl oxidase and fibronectin in breast cancer [225].

### 5.3. Cancer-Stem-Cell Regulation

Cancer stem cells (CSCs) represent a subpopulation of tumor cells with unique cell-cycle dynamics that are essential for their tumor initiation, recurrence, and treatment resistance. Some specific miRNAs target regulatory proteins of CSCs, involved in the cell cycle, and modulate proliferation and quiescence. It has been reported that miR-338-5p is involved in regulating the cell cycle of gastric cancer stem cells (GCSCs). The population of GCSCs undergoing G0/G1 phase is significantly diminished by the overexpression of miR-338-5p, while this miRNA augments their population in S and G2/M phases. The change in the cell cycle thus offers significant insight into the potential of miR-338-5p in GCSCs as targets for therapeutic approaches against these cells [229].

In HCC cell lines Huh7 and HepG2, it is reported that miR-302a/d inhibition blocks the cell-cycle progression and the suppression of this miRNA expression leads to G1-phase arrest or delays progression to S phase. The cell-cycle initiation of liver cancer SCs is suppressed by miR-302a/d that targets E2F7. However, the upregulation of miR-302a/d suppresses the proliferation of liver cancer SCs with impeded cell-cycle transition from G1 to S phase, by the inhibition of the AKT/β-catenin/CCND1 signaling pathway [230].

In another study, it is reported that miR-449b blocked the G1 to S phase cell-cycle transition, while the inhibition of this miRNA reduced the cell population in G1 phase. miR-449b also changes the CRC-SCs cell cycle by targeting the 3′UTR regions of E2F3 and CCND1. CCND1 is a significant cyclin D1 family component that functions as the major regulator of the G1/S checkpoint [231]. In proliferating cells, E2F3 activates the S phase, as it possesses DNA-binding activity [232].

In cervical cancer cells, miR-92a-3p expression is enhanced and this facilitates cell-cycle progression in cervical cancer SCs by targeting large tumor suppressor kinase 1 (LATS1). LATS1 acts as a significant component of the Hippo signaling pathway. The upregulation of LATS1 diminishes cervical cancer SC proliferation, promoting an increased number of cells in G1 phase and a smaller number of cells in S phase. LATS1 reduces the cyclin E expression while upregulating p27 expression. Cyclin E is significant for the G1/S transition, and the association of p27 and cyclin E impedes the kinase activity, and the cell-cycle transition is negatively regulated. The upregulated expression of miR-92a-3p diminishes LATS1 that results in overexpression of cyclin E and facilitates the G1 to S phase transition, enhancing cell-cycle progression and fostering tumoral behavior in CCSCs [233].

## 6. Therapeutic Targeting and Diagnostic/Prognostic Applications of miRNAs in Cancer

miRNAs play a significant role in cancer diagnosis, prognosis, and therapy as they regulate oncogenes and tumor-suppressor genes. Therapeutic strategies targeting tumor-suppressive miRNAs and oncomiRs aim to restore normal gene expression and inhibit tumor progression. Additionally, miRNAs serve as promising diagnostic and prognostic biomarkers because of their altered expression patterns in cancer tissues and body fluids. Their stability, specificity, and potential for early cancer detection make them valuable tools for developing personalized cancer management strategies.

### 6.1. Innovative Approaches to Targeting Tumor-Suppressive miRNAs and OncomiRs in Cancer Cells

For cancer management, miRNA-based therapies are broadly classified as: (a) miRNA inhibition therapy, where antagonists suppress the overexpression of miRNAs of interest, and (b) replacement or miRNA mimics therapy, where the dysfunctional or downregulated miRNA is restored by using synthetic oligonucleotides. Thus, the synthetic oligonucleotides or miRNA agonists mimic the normal miRNAs [234].

Anti-miRNA oligomers (AMOs) or miRNA sponges, antagomirs, and miRNA masking antisense oligonucleotides inhibit the activity of cancer-promoting miRNAs [234]. Antagomirs or AMOs possess a sequence complementary to endogenous miRNAs, and this is the principal method for inhibiting miRNA function. Antagomirs or antisense oligonucleotides (ASOs) are single-stranded antisense oligonucleotides (17–22 nucleotides long) which bind to endogenous miRNAs and promote their degradation by preventing RISC processing [235,236]. Oncogenic miRNAs are targeted by antagomirs, which are considered a treatment for cancer. In pancreatic cancer cells, ASOs were used to target miR-21 and miR-221 to promote the upregulation of PTEN, reversion-inducing cysteine-rich proteins with Kazal motifs (RECK) and CDKN1B genes, known as tumor suppressors, with enhanced apoptosis and minimal proliferation [237]. Furthermore, ASOs also promote gemcitabine sensitization for PDAC cells [238].

AMOs are chemically modified by the addition of 2′-O-methyl (2′-OMe) and 2′-O-methoxyethyl (2′-MOe) groups at the 5′-end of the molecule and locked nucleic acid (LNA) to improve their stability and delivery [239]. In this regard miR-21, a well-recognized oncomiR, has been modified by a 2′-OMe group and targeted in glioblastoma and BC cell lines [240]. However, 2′-MOe modified antagomirs are superior to 2′-OMe analogs, with better binding and target specificity to mRNAs, thus improving the therapeutic process [234].

LNAs are a class of antisense molecules characterized by ribose sugar modification in which the 2′-oxygen and 4′-carbon are linked by a methylene bridge, creating a bicyclic structure, providing enhanced stability and improved binding affinity [241]. As compared to original miRNA mimetics, LNAs and modified antagomirs have an enhanced half-life. LNAs were used to inhibit miR-133 in osteosarcoma, to reduce cell invasion and improve the patient’s health. The inhibition of miR-21 and miR-221 by modified ASOs led to enhanced expression of target genes such as PTEN and CDKN1B. Moreover, in lung cancer, AMOs target miRNAs like miR-21, miR-16, and miR-181a. This therapy hampers cancer growth, arrests cell-cycle progression, and enhances cell death [234].

miRNA sponges are plasmid constructs containing several binding sites for target miRNA, enabling effective sequestration of targeted miRNA [242]. After transfection, the plasmid constructs express RNA sponges, which sequester a family of miRNAs with a common seed sequence, thus blocking their activity [243]. In one study on samples from patients with NSCLC, miR-21 expression was downregulated using miRNA sponges and supported that miR-21 acts as a significant biomarker for NSCLC treatment [234]. In addition, the whole miR-181 family was inhibited in cardiac cancer cell lines using miR-181 sponge [244].

miRNA mimics or replacement therapy reestablishes normal physiological activities by mimicking the function of underexpressed or downregulated miRNA [241]. In laryngeal cancer, let-7 was found to be downregulated [241], as its loss of function led to increased RAS protein levels, implicated in certain tumors. The introduction of let-7 mimics minimized the cancer propagation in the lungs [245]. In a similar study, let-7 mimics were used to elevate apoptosis and downregulate proliferation and invasion in colon cancer and HCC [246]. Examples of different delivery vehicles of miRNAs, disease types, and signaling pathways or biological targets affected are shown in Table 7.

**Table 7:** miRNA-based therapeutic delivery strategies in cancer: delivery vehicles, cancer types, and signaling pathways or molecular targets influenced by the delivered miRNAs.

miRNA	Loading/Delivery Vehicle	Cancer Type(s)	Signaling Pathways/Targets Influenced	References
Tumor-suppressive miRNAs				
miR-34a	Neutral liposomal NPs; polymer (PEI-PEG)	NSCLC, HCC	p53 pathway downregulates *BCL-2*, *c-Met*, *SNAI1* (apoptosis and EMT regulation)	[247]
let-7 family	Lentiviral vector; polymer carriers	Lung cancer	Ras GTPase signaling; KRAS regulation	[248]
miR-145	Polyurethane-PEI NPs	Glioblastoma	Stemness factors (OCT4/SOX2); cell differentiation pathways	[247]
miR-16	Atelocollagen conjugate	Prostate cancer (metastasis model)	Targets apoptosis regulators (e.g., *BCL2* family)	[249]
miR-29b	Cationic liposome	Lung cancer	cell-cycle regulation via *CDK6*, epigenetic pathways (*DNMT3B*)	[250]
miR-7	Cationic liposome	Lung cancer	EGFR/MAPK pathway regulators (*IRS-1*, *RAF-1*)	[251]
miR-200c	Ionizable liposomes	Lung cancer	Redox and stress response (*PRDX2*, *Nrf2* axis)	[247]
Oncogenic miRNAs				
miR-155	LNA anti-miR; pHLIP-conjugates	Lymphoma, liver, lung, pancreas, glioma	STAT3, NF-κB, RhoA, SOCS1; inflammation and proliferation pathways	[19]
miR-10b	LNA antimiRs	Breast cancer, glioblastoma	Metastasis regulators (e.g., *HOXD10*)	[252]
miR-221	Cholesterol-conjugated antimiRs	HCC	Cell cycle and apoptosis (targets CDKN1B, RB1); angiogenesis modulation	[253]
miR-21	Anti-miR oligonucleotides/nanocarriers (e.g., PAMAM)	Broad solid tumors (breast, lung, colon, pancreas)	Ras/MAPK, PI3K/Akt; affects PTEN, PDCD4, RECK tumor suppressors	[254]

### 6.2. Applications of miRNAs as Diagnostic/Prognostic Biomarkers for Cancer

For the clinical diagnosis of cancer, tissue specimens are usually used to assess the prognosis and evaluation of molecular changes. However, intratumoral heterogeneity can introduce bias into the analysis of different cancer tissue samples. In addition, several factors make it challenging to obtain the cancer tissue samples. To overcome the limitations associated with conventional tissue biopsy, liquid biopsy has emerged as an innovative approach for cancer detection [255]. Liquid biopsy involves the isolation and analysis of exosomes which are secreted from all types of cells and exist in biological fluids, particularly blood. Liquid biopsy offers a minimally invasive approach, allowing repeated sample collection, and may enable early cancer identification. Cancer-derived exosomes are promising to reveal the genetic or phenotypic alterations in cancer tissues [192]. Cancer patients often exhibit elevated exosome release, making exosome-associated biomarkers promising candidates for cancer detection [256].

As it is now clear that most human cancers present dysregulated miRNA expression, thus cancer-derived exosomes directly reflect the dysregulated miRNA expression from originating cancer cells. Several cancers of the liver, breast, stomach, ovary, colon, and esophagus present enhanced expression of miR-21 in circulating exosomes derived from plasma samples. Furthermore, bladder and prostate cancer patients show enhanced levels of miR-21 in exosomes derived from their urine samples [257]. Besides the enhanced level of miR-21 in cancers like HCC, some other miRNAs can also be found in exosomes with enhanced expression [258]. However, in contrast to enhanced expression of miR-21 in HCC patients, miR-638 was reported to be downregulated and negatively associated with tumor size, vascular invasion, and aggressiveness [259].

Combining multiple exosomal miRNA detection may improve cancer diagnosis, prognosis, and prediction of therapeutic response. For example, elevated exosomal miR-146a-5p has been associated with cisplatin sensitivity, whereas increased miR-425-3p and miR-96 levels have been linked to cisplatin resistance in lung cancer [260]. In addition, exosomes obtained from breast cancer patients showed enhanced levels of miR-301 and miR-155, correlated with neoadjuvant therapy response [261]. Elevated serum exosomal miR-301, miR-155, and miR-339-5p have been associated with resistance to gemcitabine in pancreatic ductal adenocarcinoma, response to neoadjuvant therapy in breast cancer, and responsiveness to preoperative radiotherapy in locally advanced esophageal squamous cell carcinoma, respectively [261]. Furthermore, serum exosomal miR-718 was reported to be inversely correlated with HCC recurrence, highlighting the potential of exosomal miRNAs for personalized treatment and the monitoring of posttreatment disease [262].

Although circulating exosomal miRNAs show growing promise as novel cancer markers of diagnosis and prognosis, their clinical applications require careful validation and interpretation. Despite their diagnostic potential, detection of exosomal RNAs in early stages of cancer is challenging because of their low abundance. For this purpose, nanoparticle-based biochips are used as these chips capture circulating EVs directly without isolation. This method enables one-step RNA detection with in situ signal amplification that may improve early cancer detection [263].

## 7. Multiomics Investigation of miRNA-Regulated Signaling Networks

To obtain a comprehensive regulatory landscape, recent research on multiomics approaches to study miRNAs and signaling pathway dysregulation has moved away from single-layer analysis and towards integrative, systems-level frameworks that combine transcriptomics, small RNA sequencing, epigenomics, proteomics, and increasingly metabolomics [264]. These studies identify inversely correlated regulatory pairs that converge on signaling cascades like PI3K/AKT, MAPK, TGF-β, Wnt/β-catenin, and JAK/STAT by integrating miRNA expression profiles produced by small RNA-seq with mRNA-seq data to create miRNA-mRNA interaction networks [265] (Table 8).

Researchers can ascertain if miRNA dysregulation is caused by upstream epigenetic modifications and how these changes spread via transcriptional programs to reshape signaling outputs by integrating DNA methylation and chromatin accessibility datasets [266]. Proteomic integration enhances biological accuracy beyond transcript-level prediction by confirming whether predicted miRNA-mediated repression results in changed protein abundance inside pathway nodes [267].

As researchers reconstruct multilayer regulatory networks that link miRNAs, TFs, target genes, and protein–protein interaction (PPI) modules into unified signaling maps, network biology has emerged as a key component of this integrative approach [77]. These methods uncover hub miRNAs with high topological centrality that coordinate whole signaling modules instead of concentrating on specific targets. This frequently reveals feedback and feed-forward loops that enhance or buffer pathway activity in disease situations [268]. Such network-based analyses often reveal that dysregulated miRNAs in cancer and inflammatory disorders rewire interconnected pathways rather than acting in isolation, contributing to phenotypes like EMT, immune evasion, metabolic reprogramming, and therapeutic resistance [269].

Recent developments in single-cell and spatial multiomics technologies have enabled the direct assessment or computational inference of miRNA activity within distinct cellular populations residing in complex and heterogeneous tissues [270]. By combining single-cell RNA sequencing datasets with advanced miRNA target prediction algorithms, investigators can reconstruct cell-specific regulatory networks and uncover how aberrant signaling pathways vary across tumor cells, stromal components, and infiltrating immune cell populations [271].

Advanced machine learning-based computational frameworks, including graph neural networks and latent factor modeling approaches, are increasingly being applied to integrate diverse multiomics datasets and identify biologically meaningful miRNA-pathway interactions [272]. These artificial intelligence (AI)-driven methodologies facilitate biomarker discovery, support the classification of disease subtypes, and enhance the prediction of treatment outcomes by capturing complex nonlinear regulatory networks that may not be detected through traditional correlation-based analyses [273].

Experimental validation approaches have increasingly complemented computational analyses, with CRISPR-based perturbation systems coupled to transcriptomic profiling enabling the causal evaluation of predicted miRNA-mediated signaling networks [274]. These perturbation-driven sequencing methodologies facilitate the monitoring of downstream pathway remodeling following the disruption of specific genes or regulatory elements at single-cell resolution, thereby providing mechanistic evidence to support computationally derived predictions [275].

Collectively, contemporary multiomics miRNA research is increasingly focused on dynamic and multilayered regulatory frameworks that elucidate how miRNA dysregulation reprograms signaling networks across diverse molecular levels and cellular environments. These approaches facilitate the connection of genomic and epigenomic perturbations with downstream phenotypic manifestations in complex diseases [276]. A tabular summary of the major multiomics methodologies, the biological data layers integrated, the analytical frameworks employed, and their utility in the identification and characterization of signaling pathway abnormalities is presented in Table 8.

**Table 8:** Overview of contemporary multiomics strategies used to investigate miRNA-mediated signaling pathway dysregulation.

Multiomics Approach	Data Layers Integrated	Analytical Strategy	Application to Signaling Pathways	References
miRNA-seq + mRNA-seq integration	Small RNA transcriptomics + whole transcriptome	Correlation analysis, target prediction, inverse expression filtering	Identification of miRNA-regulated PI3K/AKT, MAPK, and JAK/STAT modules in cancer and neurodegeneration	[276]
miRNA + DNA methylation + mRNA	Epigenomics + transcriptomics	Multilayer regression models, causal inference	Epigenetically driven miRNA dysregulation affecting receptor tyrosine kinase and metabolic signaling	[277]
miRNA + Proteomics	Small RNA-seq + mass spectrometry	Network reconstruction with protein-level validation	Confirmation of pathway node repression at the protein level	[278]
Network-based multiomics modeling	miRNA, mRNA, TF networks, PPI databases	Graph theory, centrality analysis	Identification of hub miRNAs controlling immune and inflammatory signaling	[279]
Single-cell multiomics	scRNA-seq + inferred miRNA activity	Cell-type-specific regulatory modeling	Mapping signaling heterogeneity within TME	[270]
AI/ML multiomics integration	Multilayer omics datasets	Graph neural networks, deep learning classifiers	Disease subtype classification and pathway module prioritization	[280]
CRISPR-perturbation + transcriptomics	Functional genomics + RNA-seq	Perturb-seq and causal modeling	Validation of predicted miRNA-regulated signaling circuits	[281]

## 8. miRNA-Based Clinical Trials for Cancer Management

miRNA-based therapeutic approaches have shown considerable promise across a variety of diseases, particularly advanced malignancies [282]. Several miRNA-targeted agents are currently progressing through different stages of clinical trials for the management of different cancers [283]. The clinical trials involving miRNA-based therapeutics include the use of miRNA mimics or miRNA replacement approach and miRNA inhibition methods.

### 8.1. miRNA Replacement

The tumor-suppressive miR-15/16 family is frequently downregulated in several malignancies, including lung and colorectal cancers that commonly metastasize to the liver [284]. Reduced expression of miR-15/16 promotes multiple metastatic processes, such as tumor growth, angiogenesis, EMT, and the maintenance of CSCs [283]. Preclinical studies have demonstrated that miR-16 mimic replacement therapy can safely suppress tumor growth and metastatic spread in malignant pleural mesothelioma (MPM) and NSCLC xenograft models [285].

To facilitate targeted delivery, TargomiRs were developed, loaded with miR-16 mimics [285]. In a Phase I clinical trial initiated in 2014, TargomiRs exhibited an acceptable safety profile in 26 patients. Among the 22 evaluable participants, one achieved a partial response, while 15 experienced stable disease, highlighting the therapeutic potential of this approach [286].

Another extensively investigated tumor-suppressive miRNA is miR-34a, which negatively regulates several oncogenic pathways by targeting genes such as MET, MYC, platelet-derived growth factor receptor α (PDGFR-α), cyclin-dependent kinase 4/6 (CDK4/6), and B-cell lymphoma 2 (BCL2) [287].

Both in vitro and in vivo studies have shown that miR-34a mimic therapy inhibits tumor growth, migration, invasion, and metastatic progression [288]. Enhanced antitumor effects have also been reported following the combined administration of let-7 and miR-34 in multiple NSCLC cell lines and in the KrasLSL-G12D/+; p53flx/flx murine model of NSCLC [289]. Subsequent investigations employed the lipid-based delivery platform NOV340 to improve systemic delivery of miR-34a mimics [289]. These findings led to the development of MRX34, a liposomal formulation of a synthetic double-stranded miR-34a mimic, which entered a Phase I clinical trial involving patients with HCC and unresectable liver metastases [289,290]. However, despite encouraging preclinical results, the trial was terminated because of severe immune-related toxicities, preventing progression to Phase II evaluation (NCT01829971, NCT02862145) [290].

miR-193a-3p is another tumor-suppressive miRNA frequently downregulated in many cancers, including HCC, NSCLC, and triple-negative breast cancer (TNBC) [291,292]. The broad antitumor activity of miR-193a-3p is attributed to its regulation of multiple oncogenic targets, including KRAS, Erb-B2 (ERBB), and S6K2 in lung cancer, PLAU (plasminogen activator urokinase) in bladder cancer, MCL-1 (myeloid cell leukemia-1) in glioma, CCND1 in prostate cancer, RAB27B in osteosarcoma, and SRSF2 (serine and arginine rich splicing factor 2) in HCC [293,294].

It is reported that miR-193a-3p mimics suppress cancer cell growth and survival through the induction of cell-cycle arrest, apoptosis, cellular senescence, and DNA damage [295]. Based on these findings, integrin beta 3 (INT-1B3), a miR-193a-3p mimic encapsulated within a lipid nanoparticle (LNP) delivery system, was developed as a therapeutic candidate [296]. In preclinical tumor-bearing mouse models, systemic administration of INT-1B3 produced significant antitumor effects [296]. Consequently, a Phase I/Ib clinical trial is currently evaluating the safety, pharmacokinetics, pharmacodynamics, and preliminary efficacy of INT-1B3 in patients with advanced solid tumors (NCT04675996) [296].

### 8.2. miRNA Inhibition

The oncogenic miR-221/222 is frequently overexpressed in a variety of solid malignancies, including lung cancer [297], breast cancer [298], HCC [299], and glioblastoma [300], as well as multiple myeloma [301]. In advanced tumors, elevated miR-221 expression suppresses several tumor-suppressive targets, including p27, p57, PUMA, and PTEN, thereby promoting tumor progression and growth [296]. A significant antitumor activity of anti-miR-221 LNA-based inhibitors was observed in both in vitro and in vivo models [301]. A Phase I clinical trial (NCT04811898) is evaluating intravenous administration of LNA-i-miR-221 in patients with multiple myeloma and advanced solid tumors.

Another extensively studied oncogenic miRNA, miR-155, plays a key role in immune regulation and is aberrantly expressed in numerous solid and hematological malignancies, including breast, lung, and liver cancers, as well as leukemia [302]. Increased miR-155 expression has been associated with activation of the JAK/STAT, NF-κB, and PI3K/AKT signaling pathways, contributing to tumor cell survival and T-cell receptor-mediated responses [303]. Inhibition of miR-155 has been shown to suppress cellular proliferation and promote apoptosis in T-cell lymphoma models [303]. Furthermore, systemic delivery of LNA-based anti-miR-155 agents in B-cell lymphoma xenografts significantly reduced tumor burden and metastatic spread [304]. The use of anti-miR-155 molecules with peptide nucleic acid backbones showed promising results that facilitated the development of Cobomarsen, possessing enhanced stability [303].

Clinical evaluation of Cobomarsen began with a Phase I trial initiated in 2016 in patients with cutaneous T-cell lymphoma (CTCL), particularly the mycosis fungoides subtype. The study reported clinical benefit in approximately 95% of enrolled patients, with greater therapeutic responses observed among those receiving multiple treatment cycles [303]. Encouraged by these findings, Phase II studies were launched in 2019; however, development was discontinued in 2020 for commercial reasons despite promising early clinical outcomes (NCT03603431, NCT03713320, NCT03837457).

## 9. Future Directions, Emerging Technologies, and Challenges

Looking ahead, research on miRNA-mediated crosstalk with cancer-associated signaling pathways is expected to advance significantly through emerging technologies and integrative, multidimensional approaches that have the potential to reshape both mechanistic insights and clinical applications [305]. Technological innovations, including microarray platforms, next-generation sequencing (NGS), and quantitative real-time PCR (qRT-PCR), have already facilitated comprehensive and high-throughput profiling of miRNA expression patterns. In addition, CRISPR/Cas-based genome and epigenome editing systems offer powerful tools for the precise modulation of miRNA genes and their regulatory regions, enabling the correction of aberrant signaling networks at their origin [306]. Moreover, the convergence of AI and machine learning (ML) with large-scale multiomics datasets is accelerating the discovery of patient-specific miRNA signatures and predictive biomarkers. These advances are expected to support precision medicine by guiding personalized therapeutic interventions and the development of combinational treatment strategies aimed at simultaneously targeting multiple dysregulated signaling pathways [265].

Recent advances in single-cell sequencing, spatial transcriptomics, and high-resolution multiomics technologies have made it possible to characterize miRNA expression profiles and signaling pathway dynamics with unprecedented cellular and spatial precision. These innovations facilitate the development of increasingly accurate gene regulatory network models by integrating computational and experimental approaches, thereby providing insights into complex biological processes that would be difficult to elucidate using either strategy independently. However, experimental validation of these models often necessitates the targeted manipulation of specific genes, a task that is currently feasible for a limited set of critical regulators but remains challenging when applied simultaneously across entire signaling pathways. Moreover, the complexity of miRNA-mediated regulation further complicates network reconstruction, as a single miRNA can influence numerous target genes, while multiple miRNAs may act cooperatively or redundantly to regulate the same biological pathway or cellular process.

Several miRNA-based therapeutic strategies, including miRNA inhibition and replacement approaches, have been developed and explored for cancer treatment. Despite their therapeutic potential, the clinical translation of miRNA-based therapies remains constrained by several delivery and safety challenges. Lipid nanoparticles (LNPs), commonly used to deliver therapeutic miRNAs, often exhibit preferential hepatic accumulation, which may limit their delivery to extrahepatic tumors and necessitate the development of tissue-specific targeting strategies. Furthermore, cellular uptake does not necessarily translate into therapeutic efficacy, as a substantial proportion of the delivered cargo may remain trapped within endosomes and undergo degradation before reaching the cytoplasm. Endosomal escape therefore represents a critical determinant of functional miRNA delivery.

In addition, therapeutic miRNAs and their delivery vehicles may activate innate immune pathways, including Toll-like receptor (TLR)-mediated signaling, potentially inducing inflammatory responses and compromising treatment tolerability. Another major concern is the intrinsic capacity of miRNAs to regulate multiple mRNA targets, which may result in unintended gene suppression and adverse effects in normal tissues. Addressing these limitations will require advances in tissue-selective delivery, endosomal release, immunocompatible formulations, and systematic evaluation of on-target and off-target effects. Comprehensive preclinical assessment of biodistribution, pharmacokinetics, immune activation, and toxicity will be essential for improving the safety and clinical applicability of miRNA-based therapeutics.

Nevertheless, ongoing advances in molecular biology, nanotechnology, and precision medicine are expected to accelerate the clinical translation of miRNA-based therapeutics. As a result, miRNA-targeted interventions may emerge as a vital component of next-generation cancer treatment strategies, offering innovative solutions to improve outcomes in patients with advanced and treatment-resistant malignancies. Continued investigation is therefore essential to overcome existing limitations and fully harness the potential of miRNAs as biomarkers and therapeutic targets for early diagnosis, prognostic assessment, and personalized cancer management.

## 10. Conclusions

The biogenesis of miRNAs is a complex process, and numerous factors influence miRNA expression and function. The dynamic interplay between miRNAs and major signaling pathways forms a central regulatory layer that is commonly disturbed in cancer, contributing to tumor development, progression, metastatic dissemination, treatment resistance, and the persistence of CSC populations. Aberrant miRNA expression influences key oncogenic signaling pathways including PI3K/AKT/mTOR, MAPK/RAS/ERK, Wnt/β-catenin, TGF-β/SMAD, Notch, and STAT3-associated inflammatory pathways, while also restructuring feedback and feed-forward circuits that sustain abnormal signaling and cellular plasticity. Such network-wide disruption promotes malignant characteristics, including EMT, invasion, and adaptation to the TME, and enhances the ability of cancer cells to withstand conventional chemotherapy and targeted treatments. Increasing mechanistic insight, together with advances in single-cell multiomics, spatial transcriptomics, computational modeling, and RNA-based therapeutic platforms, is facilitating the development of precision oncology strategies that target miRNA-signaling interactions at multiple regulatory points. Therapeutic approaches that restore tumor-suppressive miRNAs, inhibit oncomiRs, or regulate critical signaling hubs may reverse chemoresistance, restrict metastasis, and eliminate CSCs, ultimately improving clinical outcomes. Developing innovative strategies to selectively modulate miRNA activity, including miRNA mimics, inhibitors, and targeted delivery systems to enhance tissue specificity while minimizing off-target effects, may provide novel therapeutic opportunities for diseases associated with miRNA dysregulation. Collectively, current evidence establishes miRNA-mediated regulatory networks as major determinants of cancer biology and highlights the potential network-based interventions to enable more effective, personalized, and durable treatments across a broad range of malignancies.

## Data Availability

No new data were created or analyzed in this study. Data sharing is not applicable to this article.
